# Indian Ocean corals reveal crucial role of World War II bias for twentieth century warming estimates

**DOI:** 10.1038/s41598-017-14352-6

**Published:** 2017-10-31

**Authors:** M. Pfeiffer, J. Zinke, W.-C. Dullo, D. Garbe-Schönberg, M. Latif, M. E. Weber

**Affiliations:** 1 RWTH Aachen University, Geological Institute, Wüllnerstrasse 2, 52056 Aachen, Germany; 2 Freie Universitaet Berlin, Section Palaeontology, Malteserstrasse 74-100, 12249 Berlin, Germany; 3 Curtin University of Technology, Department of Environment and Agriculture, Kent Street, Bentley, WA 6102 Australia; 4 Australian Institute of Marine Science, PMB 3, Townsville MC Queensland, 4810 Australia; 5 University of the Witwatersrand, School of Geography, Archaeology & Environmental Studies, Wits, 2050 South Africa; 6 GEOMAR Helmholtz Centre for Ocean Research, Wischhofstrasse 1-3, 24148 Kiel, Germany; 7 Institute for Geosciences, Kiel University, Ludewig-Meyn-Str. 10, 24118 Kiel, Germany; 8 Steinmann Institute, University of Bonn, Poppelsdorfer Schloss, 53115 Bonn, Germany

## Abstract

The western Indian Ocean has been warming faster than any other tropical ocean during the 20^th^ century, and is the largest contributor to the global mean sea surface temperature (SST) rise. However, the temporal pattern of Indian Ocean warming is poorly constrained and depends on the historical SST product. As all SST products are derived from the International Comprehensive Ocean-Atmosphere dataset (ICOADS), it is challenging to evaluate which product is superior. Here, we present a new, independent SST reconstruction from a set of *Porites* coral geochemical records from the western Indian Ocean. Our coral reconstruction shows that the World War II bias in the historical sea surface temperature record is the main reason for the differences between the SST products, and affects western Indian Ocean and global mean temperature trends. The 20^th^ century Indian Ocean warming pattern portrayed by the corals is consistent with the SST product from the Hadley Centre (HadSST3), and suggests that the latter should be used in climate studies that include Indian Ocean SSTs. Our data shows that multi-core coral temperature reconstructions help to evaluate the SST products. Proxy records can provide estimates of 20^th^ century SST that are truly independent from the ICOADS data base.

## Introduction

Surface warming of the Indian Ocean is larger than the global average SST rise^1,2^. Maximum warming occurs in the central equatorial region, along the so-called Seychelles-Chagos thermocline dome^2,3^, and appears to be caused by a decrease of upwelling-related oceanic cooling in addition to anthropogenic forcing^2,4^. The reduced upwelling may result from a weakening of the summer monsoon cross-equatorial flow in recent decades^2^.

**Figure 1:**
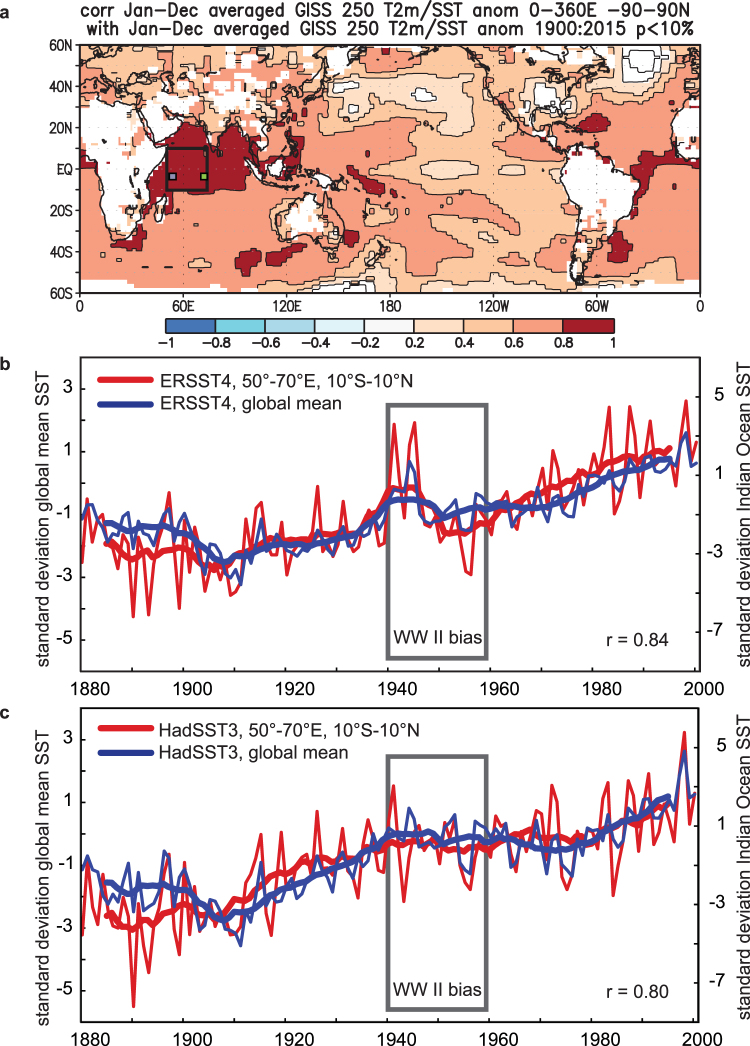
Historical Indian Ocean and global mean temperature. (**a**) Field correlation of the global mean temperature record with gridded temperature data (GISSTEMP 250 km resolution, blended surface air temperature and SST)^32^. Note the high correlation in the tropical Indian Ocean. The black rectangle indicates the target region of our study: the western tropical Indian Ocean (10°N-10°S, 50°E-70°E). The small rectangles indicate the location of the Seychelles (green) and the Chagos Archipelago (blue). Correlations not significant at the 10% level are masked out. Correlations computed using the free web application KNMI Climate Explorer (available at http://climexp.knmi.nl/) and plotted with GrADS 2.0 (free software; http://cola.gmu.edu/grads/). Date accessed: April 6, 2017. (b) Instrumental SST of the western tropical Indian Ocean extracted from the ERSST4 product^9^ (thin blue line: annual means; thick blue line 11 point moving averages) compared with global mean ERSST4 data (thin red line: annual means; thick red line: 11 point moving averages). (c) Same as B but for HadSST3^7,8^. In each case, western Indian Ocean and global mean SST are highly correlated (annual means: r = 0.84 and 0.80, respectively, which is significant at the 1% level assuming 118 degrees of freedom, n = 120) and the 20^th^ century Indian Ocean warming is proportional to the global mean SST trend. However, the temporal pattern of the warming depends on the SST product. Discrepancies are largest during and after the World War II period (1940–1960, black bar).

SST over the Indian Ocean is strongly linked to global mean temperature (surface air temperature and sea surface temperature combined) and varies in-phase with it^1,5^ (Fig. 1a,b; Supplementary Fig. S5). Although Indian Ocean warming has large global relevance^1,4,5^, there are significant discrepancies between various SST datasets regarding the temporal pattern of this warming during the 20^th^ century (Fig. 1 b,c; Supplementary Fig. S5). Differences between SST products are largest during and after World War II (WW II) and appear as a warm anomaly followed by an abrupt cooling after 1945 in some SST products (Fig. 1b; Supplementary Fig. S5), while others show a steady warming during the first half of the 20^th^ century followed by a slight cooling trend that ends with a shift towards warmer temperatures in the mid-1970s (Fig. 1c; Supplementary Fig. S5). The WW II period is problematic as the war lead to a sudden shift in measurement techniques, causing biases in the global SST record^6^. Attempts have been made to correct these biases^7–9^, but the resulting SST products still do not agree on 20^th^ century warming trends (compare Fig. 1b,c). In the western Indian Ocean, the World War II anomalies dominate the 20^th^ century SST trend. They also appear prominently in the global mean SST record (Fig. 1b), and this should reflect an impact of the Indian Ocean on global mean SST. It is therefore of paramount importance to reconstruct with high confidence 20^th^ century western Indian Ocean warming in order to evaluate its impact on regional and global temperature variability.

Here, we present a multi-core coral SST reconstruction from the western tropical Indian Ocean. The coral sites are chosen to reflect SST variability in the Seychelles-Chagos thermocline ridge. We chose a literature-based proxy-temperature conversion that ensures that the coral temperature reconstruction is truly independent from instrumental temperature data. Thus, we can evaluate and compare 20^th^ century warming patterns in the coral and historical temperature datasets from various sources.

## Results

### Composite temperature reconstruction

**Figure 2:**
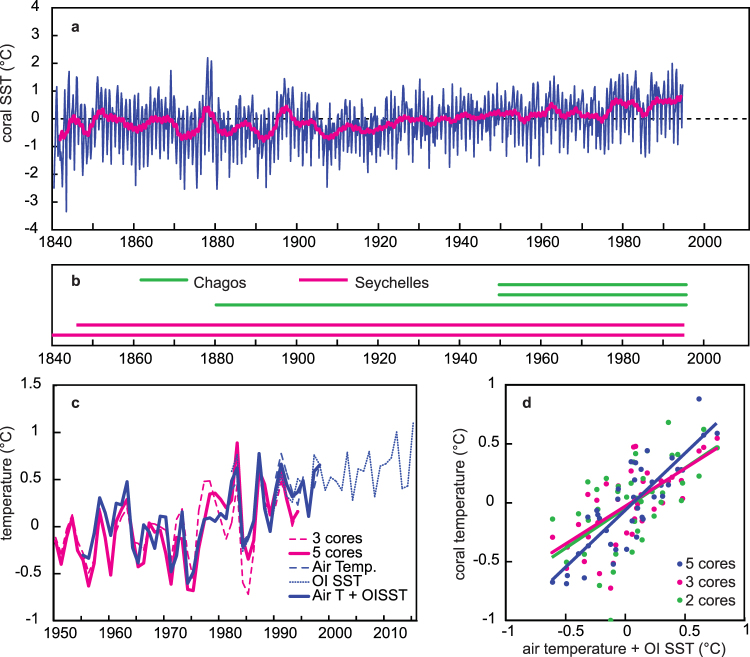
Coral temperature reconstruction: validation. (a) Composite coral temperature reconstruction for the western tropical Indian Ocean. The thin blue line are the bimonthly values, the thick magenta line is a 61-point moving average. (b) Coral cores included in the composite record. Magenta line: coral δ^18^O records from the Seychelles^10,11^, Green line: coral Sr/Ca records from the Chagos Archipelago (this study and^12,13^). (c) Comparison of the coral derived temperatures (5 and 3 core averages) with a composite record computed from local air temperature (Mahe, Seychelles, and Diego Garcia, Chagos; see supplementary material) and local OI SST (1° × 1° grid, available since 1982^18^). All records have been centered by subtracting their mean. (OI SST has been centered relative to the mean of the air temperature between 1982 and 1998) (d) Scatter plot of coral derived temperatures vs. the local air temperature/OI SST composite. For the 5-core reconstruction, the correlation is r = 0.84 (significant at the 1% level assuming 38 degrees of freedom, n = 40) and the slope of the linear regression is not significantly different from 1.

Our multi-core coral reconstruction comprises a set of *Porites* coral oxygen isotope (δ^18^O; The Seychelles, 50°E, 5°S)^10,11^ and Sr/Ca records (Chagos Archipelago, 70°E, 5°S)^12,13^ covering the past 50 to 155 years (Fig. 2 a,b, Supplementary Figs S1–S4). The Seychelles (Chagos) are located in the western (eastern) zone of the Seychelles-Chagos thermocline dome, a dome-like feature in thermocline depth in the south-western tropical Indian Ocean^2,3^. At both sites, SST variations are highly correlated and a combined temperature record (arithmetic mean) from the two sites captures large-scale SST variability in the western tropical Indian Ocean (Supplementary Figs S6, S7). The composite chronology is a bimonthly resolved time series that extends from 1840 to 1995 (Fig. 2 a,b). Well-replicated time periods are 1950–1995 (5 cores) and 1880–1995 (3 cores).

At the Seychelles, coral δ^18^O reflects primarily temperature variability. This has been documented in previous calibrations of coral δ^18^O with temperature^10,11^, as well as paired coral δ^18^O and Sr/Ca measurements of modern and Holocene Seychelles corals^14^. Seawater measurements of δ^18^O carried out at the Seychelles (Supplementary Fig. S8) and in the western Indian Ocean show that variations are smaller than the analytical uncertainty of coral δ^18^O on seasonal to decadal time scales and cannot measurably influence coral δ^18^O (see Supplementary Methods and Discussion).

At Chagos, *in situ* δ^18^O seawater data are lacking, but a calibration of coral δ^18^O suggests a measurable contribution of δ^18^O seawater^12^. Chagos lies in the center of the Intertropical Convergence Zone during boreal winter and hence rainfall is abundant. The seasonal cycle of surface salinity is 0.64 psu, which is twice as large as at the Seychelles^15^. Therefore, we developed monthly resolved Sr/Ca records in order to reconstruct sea surface temperatures at Chagos (see Methods and Supplementary material for a discussion of analytical procedures). These records have been shown to record local temperature variability^12,13^.

At both sites, the SW monsoon induces large-scale cooling during boreal summer that is recorded by the corals and can be traced into the Arabian Sea^10–14^. The corals also record El Nino-induced warming that affects the entire western Indian Ocean sector and peaks in boreal winter to spring^10–14^.

A composite coral temperature record was constructed by (1) centering all proxy records by removing the 1961-1990 mean (a base period commonly used for historical temperature data), (2) converting each proxy record to temperature units, (3) calculating the arithmetic mean of the coral records from each site, and (4) averaging the mean records from both sites. The temperature conversion is based on the known proxy-temperature relationship (−0.2‰/°C for δ^18^O^16^ and −0.06 mmol/mol/°C for Sr/Ca^17^). The main advantage of this approach is that (I) the coral temperature variations are estimated independently from local instrumental data, and (II) we can quantitatively validate the coral temperature variations with local air temperature and SST records.

Note, however, that the coral reconstruction is not sensitive to the method of construction. For example, a composite coral record derived by normalising each single proxy record to its standard deviation prior to averaging is almost identical to the composite record obtained by converting the proxy records to SST units using the published proxy-SST slopes (r = 0.97, which is significant at the 1% level assuming 138 degrees of freedom, n = 140). However, with this approach we would lose all information on quantitative temperature variations indicated by the coral proxies, which is an important check of the quality of the reconstruction.

### Validation: local temperature

We validate the coral temperature reconstruction with a composite surface air temperature record computed from the Chagos and the Seychelles weather stations (Supplementary Fig. S9) blended with the 1° × 1° OI SST data centered on each island (available since 1982)^18^ (Fig. 2 c,d). The OI SST is used to fill data gaps in the air temperature record during recent years (Supplementary Fig. S9). The coral reconstruction is qualitatively and quantitatively consistent with the temperature observations: the correlation between the two series is very high (r = 0.85 for annual means, which is significant at the 1% level assuming 38 degrees of freedom, n = 40) and the slope of the ordinary least squares (OLS) regression is not significantly different from 1. The three-core coral reconstruction has a slightly lower correlation with the surface air/satellite temperature composite (r = 0.65, which is significant at the 1% level assuming 38 degrees of freedom, n = 40). The standard deviation of the residuals increases from 0.2 °C (five core composite) to 0.24 °C (three core composite). Thus, the coral temperature reconstruction should be very robust at least until 1880, when the longest coral record from Chagos ends and only two coral records from the Seychelles remain. For this two-core reconstruction the correlation reduces to r = 0.53 (which is significant at the 1% level assuming 38 degrees of freedom, n = 40), with a standard deviation of residuals of 0.3 °C. Scatter-plots do not show a fundamentally different coral temperature – instrumental temperature relationship for the 3- and 2-core coral composites (Fig. 2 d). However, the correlation coefficient between coral temperature and instrumental temperature influences the OLS slope: a lower correlation coefficient results in a smaller slope.

Very similar results are obtained when regressing the first differenced coral temperature reconstructions (5- 3- and 2-core composite) against local air temperature (Supplementary Fig. S12, Supplementary Table S3). This confirms that our coral reconstruction provides correct estimates of interannual temperature variations. Supplementary Table S4 compares the standard deviations of the coral temperature reconstructions (5- 3- and 2-core composite) and local temperature (original and first differenced time series). The standard deviations (in °C units) estimated from the corals and local air temperature are not significantly different (based on a two sided F-test).

### Historical temperature data: World War II bias

**Figure 3:**
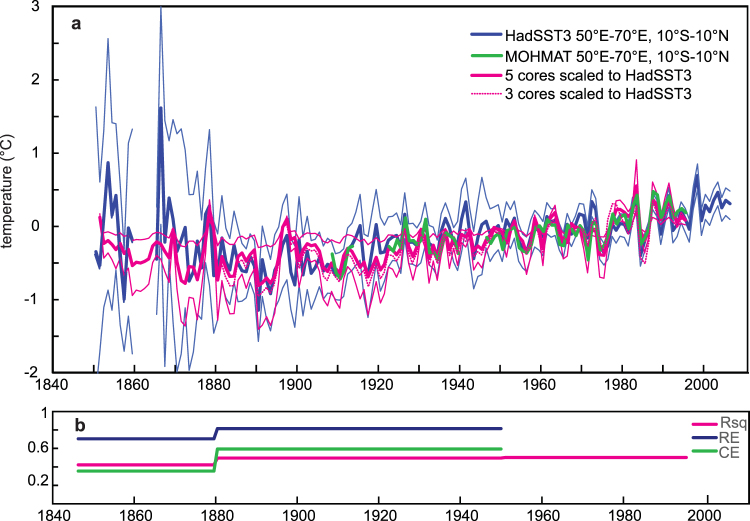
Western Indian Ocean coral temperature reconstruction. (a) Coral composite temperature reconstruction scaled to large-scale western Indian Ocean SST (WIO coral; thick magenta line: annual mean of 5-core average, thin lines: 95% CL; dashed magenta line: annual mean of 3-core average). The WIO coral reconstruction has been scaled to the standard deviation of HadSST3^7,8^ averaged over 50°E-70°E, 10°N-10°S (thick blue line: annual mean; thin lines: 95% CL; see supplementary methods); base period: 1961–1990. The WIO coral temperature reconstruction is consistent with MOHMAT4^19^ (thick green line) averaged over 50°E-70°E, 10°N-10°S. (b) Reconstruction skill statistics of the WIO coral SST against HadSST3^7,8^ are calculated for the 5 (Rsq, magenta), 3 and 2 core composites (Rsq, magenta, reduction of error, RE, blue; coeefficient of efficiency, CE, green). Calibration period: 1950–1995; Validation period: 1908–1913, 1922–1939.

We compare the coral SST reconstruction with large-scale temperature variability averaged over the western tropical Indian Ocean (50°E-70°E, 10°N-10°S), as portrayed in various historical data products. This region displays maximum warming trends^1^ and includes the Seychelles-Chagos thermocline dome^2,3^. The large-scale temperature averages have lower standard deviations than the coral temperature reconstructions (Supplementary Table S5). Therefore, we need to scale the coral reconstruction to the regional temperature averages. We chose HadSST3 as the standard series to adjust the coral data (Fig. 3, Supplementary Fig. S10). The coral reconstruction is scaled to the standard deviation of HadSST3 for the base period of 1961–1990 (n = 29). The adjusted western Indian Ocean (WIO) coral temperature reconstruction is shown in Fig. 3 a. The uncertainty of the WIO coral SST reconstruction is calculated from the 95% confidence intervals of the HadSST3 and coral standard deviations between 1961 and 1990 (thin red lines in Fig. 3 a). Figure 3 b shows the coefficient of determination (R^2^) for the 5-, 3- and 2-core WIO coral reconstruction (calibration data: HadSST3, 1950–1990). Reconstruction skill statistics (reduction of error, RE and coefficient of efficiency, CE) are computed for the 3- and 2-core reconstruction (HadSST3, Calibration period: 1950–1995; validation period: 1908–1913 and 1922–1939; Fig. 3 a,b). The validation period is restricted to well-observed time periods, when the number of observations in HadSST3 is large (Supplementary Fig. S11). Although reconstruction skill decreases as the number of corals decreases, we find that even the 2-core WIO coral record comprising only Seychelles corals has statistical skill for a western Indian Ocean SST reconstruction (Fig. 3 b). The decrease in the number of coral cores may affect the variance of the WIO coral reconstruction, as the 3- and 2-core reconstructions have slightly lower standard deviations than the 5-core composite (Supplementary Table S4). However, this effect is small, especially in the mid-20^th^ century when the variance of the coral reconstruction is small. Figure 3 a shows the 3-core reconstruction scaled to SST according to its standard deviation for comparison. Supplementary Fig. S13 compares the 5- and 3-core reconstructions with western Indian Ocean HadSST3 after removing the linear trends.

Results are also compared with MOHMAT 4^19^ averaged over the western tropical Indian Ocean (50°E-70°E, 10°N-10°S) (Fig. 3). MOHMAT 4 is an older version of night-time marine air temperature data. However, a recent study has shown that MOHMAT 4 spatial trend patterns are consistent with other independent estimates of Indo-Pacific climate change (sea level pressure, surface winds and bucket-only SSTs from ICOADS)^20^. This suggests that, unlike the SST products, MOHMAT 4 is not affected by instrumental biases or other quality issues associated with data coverage, and therefore ideal to assess long-term trends^20^. Missing data is not interpolated, and there are large data gaps in the 50°E-70°E, 10°N-10°S region (Fig. 3).

To assess the influence of the WW II bias on the warming trend in the western Indian Ocean, we compare the WIO coral temperature reconstruction with various historical SST products. We extract the relevant grid-data from the newest historical products with WW II bias correction (ERSST 4^9^ and HadSST3^7,8^) and compare them with their previous versions, which are lacking bias corrections between 1940 and 1960 (ERSST3b^21^ and HadSST2^22^, respectively). As an independent check for our coral reconstruction, we use recent gridded products of night-time marine air temperature (NMAT) data. NMAT data is independent from SST and less affected by changes in observational methods. We use HadNMAT2^23^ and HadMAT1^19^ (newest and previous version, respectively).

**Figure 4:**
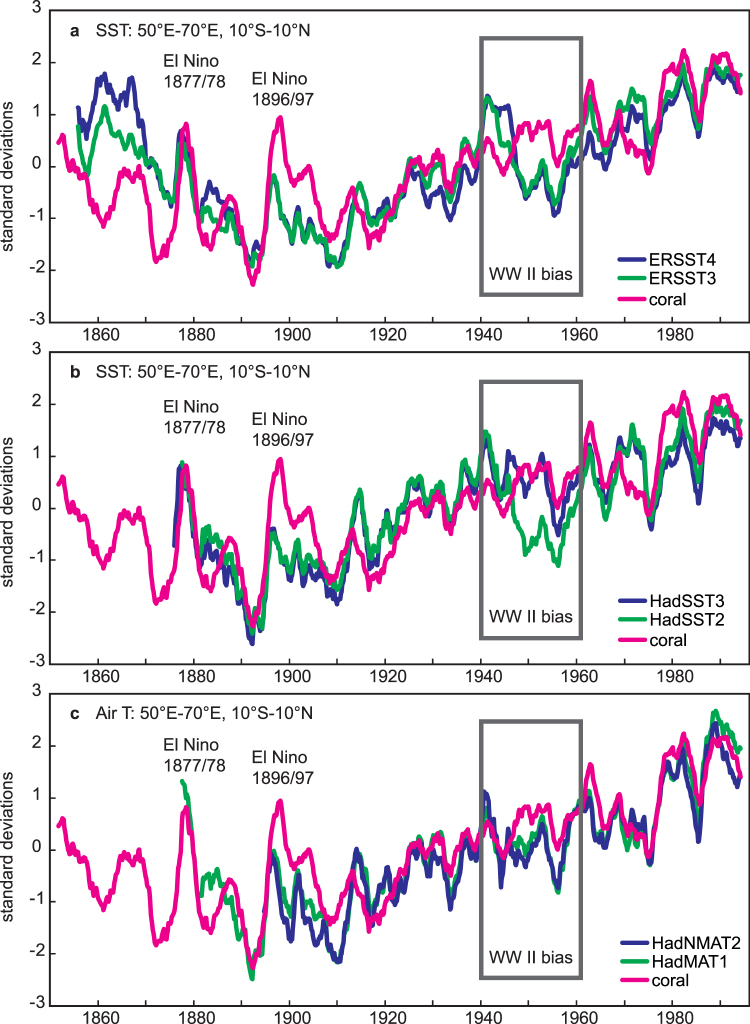
Western Indian Ocean coral and historical temperatures: WW II bias. (a) Western Indian Ocean (WIO) coral temperature reconstruction (magenta) compared with WIO ERSST3^21^ (green) and ERSST4^9^ (blue). (b) Same as A using HadSST2^22^ and HadSST3^7,8^. (c) Same as A using night-time marine air temperature (HadMAT1^19^ and HadNMAT2^23^). All time series are 21 point moving averages of bimonthly anomalies, and have been normalized to their standard deviation over the 1900–1995 period. Note different SST trend patterns during and after World War II depending on the SST product (black bar: time period of World War II bias). The WIO coral indicates large amplitude temperature anomalies during the late 19th century El Niño events of 1877/78 and 1896/97. See text for discussion.

**Figure 5:**
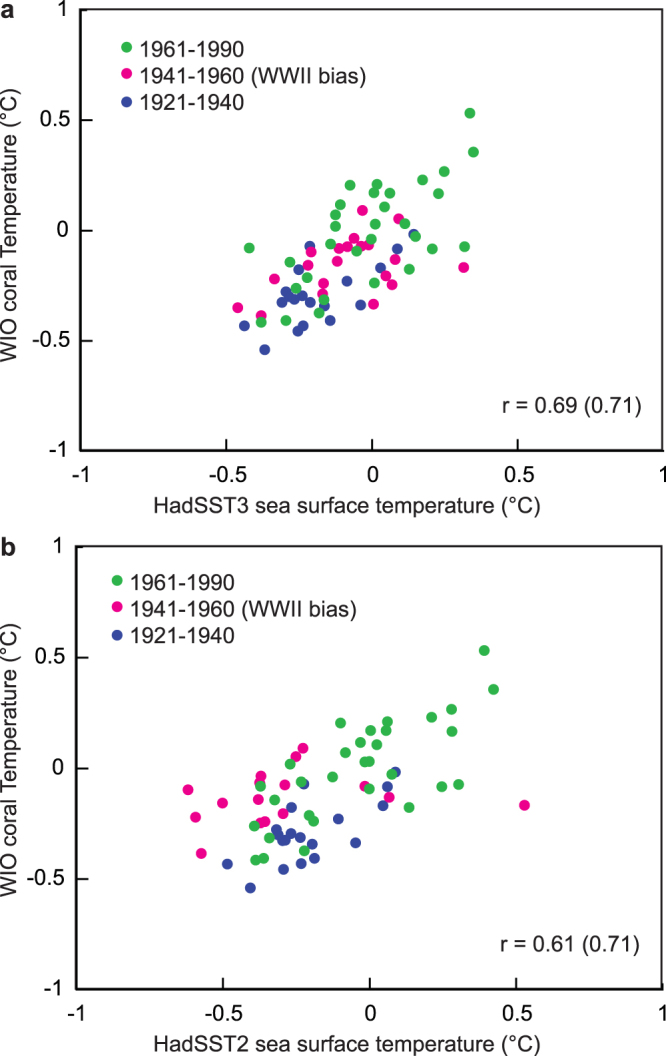
WW II bias correction and coral-temperature correlation. Scatterplots of annual mean WIO coral temperature vs. (a) WIO HadSST3^7,8^ and (b) WIO HadSST2^22^. Green dots: 1961–1990, magenta dots: 1941–1960 (WW II bias), blue dots: 1921–1940. Note the improved correlation between WIO HadSST3 and WIO coral temperature during the time period affected by the WW II bias. Correlation coefficients are calculated over the 1921–1990 period and omitting the WW II bias period from 1941–1960 (in brackets).

Figure 4 compares interannual to decadal variations of the WIO coral reconstruction with SST and night-time marine air temperature data from the western Indian Ocean. All series are 21-point moving averages of bimonthly anomalies, normalized to their standard deviations. Normalizing the data has the advantage that we can focus on the temporal pattern of the coral, SST and NMAT trends. Note that only ERSST4 and HadSST3 employ a WW II bias correction between 1940 and 1960^7–9^. Each product uses a different method for this bias correction: ERSST4^9^ uses HadNMAT2, while HadSST3 is corrected based on the measurement method (i.e. bucket or engine-room intake)^7,8^. As expected, the older versions of the SST reconstructions (ERSST3, HadSST2) show a large warm/cool shift during and after World War II (Fig. 4 a,b). This warm/cool anomaly is clearly associated with the WW II bias described previously^6^, and is not seen in the coral-based SST reconstruction. The warm/cool shift is also not seen in HadSST3 and consequently, the 20^th^ century warming pattern indicated by this SST product agrees much better with the coral record (Fig. 4 b). Scatter plots of HadSST2 and HadSST3 vs. the WIO coral record also show the improvement of HadSST3 during 1941–1960 (Fig. 5). In contrast, the WW II bias still appears in ERSST4, the newest SST product presently available, which does not look much different compared to ERSST3 (Fig. 4 a). Thus the coral reconstruction supports the HadSST3 WW II bias correction. The coral temperature trend pattern agrees well with both night-time marine air temperature products, which, as noted above, are independent from the SST data (Fig. 4 c).

The cool bias between 1945 and 1960 affects the warming rates calculated for the western Indian Ocean since 1950 – a time period frequently used in climate change studies^3,20^. The trend estimates begin in an apparently cool period and may be inflated, yielding an accelerated warming after 1950 (Fig. 4, Supplementary Table S6). Warming rates calculated over the period 1900 to 1995 are not affected by the WW II bias (Fig. 4, Supplementary Table S6).

**Figure 6:**
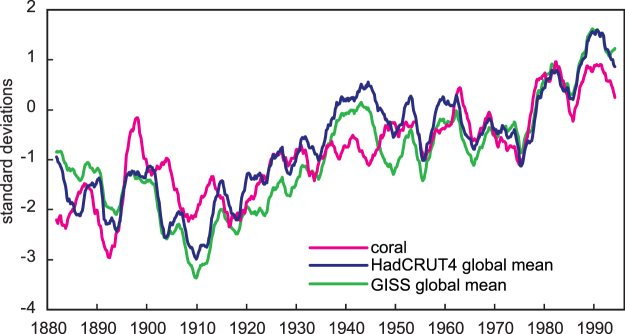
Western Indian Ocean corals and global mean temperature. WIO coral temperature reconstruction (magenta) compared with global mean temperature (blue line: HadCRUT4^33^; green line: GISTEMP 1200 km^32^). Shown are 21 point moving averages of bimonthly anomalies, normalized to their standard deviation over the 1900–1995 period. The correlation between the coral and HadCRUT4 (GISTEMP) is r = 0.82 (0.78), significant at the 1% level assuming 31 degrees of freedom (21 point average of bimonthly time series: n = 668/21, n-2 = 31). Note the good agreement between the WIO coral reconstruction and global mean temperature in the 1950–1995 period, when 5 coral cores are available from the western Indian Ocean.

As expected from the strong impact of western Indian Ocean SST on global mean SAT (Fig. 1, Supplementary Fig. S5), the WIO coral reconstruction also closely follows the long-term evolution of global mean temperature (Fig. 6). Note the excellent match between the coral and global mean temperatures between 1950 and 1995. During this time period, the coral reconstruction comprises 5 cores from the western Indian Ocean. This underlines the great potential of multi-core coral temperature reconstructions to characterize 20^th^ century SST variations.

Overall, the WIO coral SST record shows colder mean temperatures with strong inter-annual to decadal variability prior to 1920 and reduced decadal variability between 1920 and 1975. A gradual warming until the 1960s is followed by a short cooling period in the early 1970s, and a rapid shift towards higher temperatures in the mid-1970s that is also seen in the global average temperatures (Fig. 6) and has been considered real^24^. The high variability prior to 1920 partly reflects increased El Niño/Southern Oscillation (ENSO) variability during the last quarter of the 19^th^ century^25^. For example, two extreme El Niño events occurred in 1877/78 and in 1896/97^25,26^, consistent with anecdotal evidence. These “super” El Niño events appear as distinct warm anomalies in the coral record (Fig. 4). Since the mid-1970s, almost every year has been warmer in terms of full temperature than even the warmest climate extreme prior to 1900 (Figs 2, 3 and 4).

## Discussion

Sea surface temperature (SST) is among the most fundamental variables to measure climate change. The historical SST products are an important basis for a large number of studies that aim at investigating the climate’s response to greenhouse gas forcing, and they are used to force atmosphere models^3^. Uncertainties in SSTs often affect the results, especially in the equatorial Indo-Pacific^3,20,27^. Long-term warming trends are generally much smaller than the magnitude of interannual to decadal variability, and are therefore much more sensitive to time-varying biases in the instrumental records^20,27^. Our results suggest that the World War II bias owing to a switch in the technique measuring SSTs has a strong impact on the 20^th^ century warming trend in the western tropical Indian Ocean. This region is a large contributor to the overall warming trend in global mean SST and features a pronounced warming with relatively weak superimposed interannual to decadal variability^1^. The World War II bias corrections applied to the most recent SST products still results in very different temporal warming patterns in the western tropical Indian Ocean, and the same pattern is then seen in the global mean SST record (Fig. 1 b,c). This suggests that, in order to better estimate 20^th^ century warming, we need to enhance Indian Ocean SST estimates.

Our multi-coral WIO temperature reconstruction was developed using the known proxy-SST relationships, which ensures it is truly independent from instrumental SSTs. A comparison with local temperature records confirms the quantitative SST variations estimated form the coral reconstruction. In order to reconstruct WIO-averaged temperatures, the variance of the coral reconstruction has been adjusted. Calibration/validation exercises suggest that the adjusted WIO coral reconstruction has statistical skill until 1847 (after that time, only one coral record from the Seychelles remains). The 20^th^ century temperature trend portrayed by the coral composite is consistent with HadSST3 (and night-time marine air temperature data), and suggests that the latter should be used preferably in climate studies that include Indian Ocean SSTs. Previous studies have shown that the 50-year trend of the Indo-Pacific Walker Circulation is highly controversial^3^. From a set of SST products, only HadSST3 shows a weakening of the Indo-Pacific Walker Circulation since 1950, which would be consistent with sea level pressure measurements of the 20^th^ century^3,27^. This example nicely illustrates the importance of correct SST estimates during and after WW II.

The new coral SST reconstruction also suggests pronounced interdecadal variability in the tropical Indian Ocean, with increased interannual to decadal variability prior to 1920 and again after 1975. The large-amplitude and prolonged warm SST anomalies recorded during the strong late 19^th^ century El Niño events of 1877/78 and 1896/97 appear proportionally larger compared to modern El Nino events (e.g. 192/73, 1982/83), which are also recorded by the corals^10–14^. However, they confirm the important role of El Niño for Indian Ocean warming^1^. Each of these two late 19^th^ century El Niños caused record droughts and famines in India due to a failure of the Indian summer monsoon, as well as severe droughts elsewhere^26,28^. There is nonlinearity such that the western Indian Ocean warming during El Niño events has a much larger amplitude than the cooling during La Niña events, so frequent strong El Niño events may cause prolonged warm periods in this region^1^. The recent warming of the western Indian Ocean is therefore attributed to the observed increase in the frequency and amplitude of El Niño events during the recent decades^1^. At the same time, the warm Indian Ocean has been shown to contribute to a decrease in the Indian summer monsoon by reducing the land-ocean thermal gradient^29^. At present, it is not clear if and how the western Indian Ocean contributed to the extreme droughts in the circum-Indian Ocean in the late 19^th^ century. However, given the magnitude of the warming during the late 19^th^ century El Niño events, it indeed may have affected the land-ocean thermal gradient and in turn the monsoon. Seasonal-scale coral proxy data indicate that the warming during these events started relatively early, with visible warm anomalies already during boreal summer (June-August), i.e. several months before the peak of El Niño-related warming of the WIO is reached in the following boreal spring^1^.

Significant efforts should be made to estimate and correct instrumental SSTs during and after the WW II period, particularly in the tropical Indian Ocean. Our data shows that multi-core coral temperature reconstructions help to validate these datasets. We therefore recommend that additional multi-core coral reconstructions should be developed from a number of key sites in the tropical oceans. Only proxy records can provide estimates of 20^th^ century SST that are truly independent from instrumental measurements (i.e. ICOADS). In addition, the late 19^th^ century appears a very important period for further investigation using high-resolution climatic archives, as large interannual to decadal variability (in a colder mean climate) caused devastating droughts in the countries surrounding the Indian Ocean^26,28^. A dense network of proxy data from the Indo-Pacific that includes SST reconstructions may help to identify the climatic processes that caused these catastrophic famines and could provide information’s on their likely recurrence in the future.

## Materials and Methods

### Seychelles chronology

The Seychelles chronology comprises one monthly resolved δ^18^O record^10^ that extends from 1847 to 1995, and a bimonthly δ^18^O record that extends from 1840 to 1994^11^ (Supplementary Figs S1 and S2). The two records were centered by removing the mean of the 1961–1990 time period, and converted to temperature using the δ^18^O-SST relationship of −0.2 per mill per 1 °C^16^. Both cores were then averaged to produce a bimonthly resolved time series.

### Chagos chronology

The Chagos chronology comprises three monthly resolved Sr/Ca records (Supplementary Figs S3 and S4)^12,13^. Two records extend from 1950 to 1995, and one record extends from 1880 to 1995. Only the recent 50 years of this record have been published previously^12,13^. All cores were centered by removing the mean of the 1961 to 1990 time period, and converted to temperature using the Sr/Ca-SST relationship of −0.06 mmol/mol per 1 °C^17^. The three Sr/Ca records were then averaged to produce a monthly resolved time series, and reduced to bimonthly resolution to match the Seychelles data.

### Composite chronology

Between 1880 and 1995, the composite coral temperature reconstruction is the arithmetic mean of the Chagos and Seychelles chronologies. This approach was chosen because a ‘simple’ average computed from the two SST-grids including the Seychelles and Chagos (without applying any weights to one of the grids) provides the best estimate of Western Indian Ocean SST in the region 10°N-10°S, 50°E-70°E (Supplementary Figs S6 and S7). The main advantage of this approach is that (I) the coral temperature variations are estimated independently from local instrumental data, and (II) we can quantitatively validate the coral temperature variations with local air temperature and SST records.

The most robust reconstruction covers the time period from 1950 to 1995, when five cores are available (2 from the Seychelles, 3 from Chagos) (Figs 2, 3, Supplementary Fig. S13). However, the three-core reconstruction (2 from the Seychelles, 1 from Chagos, 1880–1995) is also robust (Figs 2, 3, Supplementary Fig. S13).

### Statistics

For consistency, all time series (coral and instrumental) are reduced to bimonthly resolution. Annual average values are calculated from the bimonthly data. To determine the accuracy of the large-scale WIO temperature reconstruction, we calculated the 95% confidence interval in the spread of the SST standard deviation for 1961–1990 in both WIO coral SST and HadSST3 and subsequently estimated the spread in the scaling coefficients following^30^. We used the maximum spread in the scaling coefficients as uncertainty bounds on our final WIO coral SST record (thin red lines in Fig. 3A). To allow for the number of coral records decreasing backwards in time, reconstruction skill statistics^31^ were calculated over the validation period 1908–1913 and 1922–1939 for the 2 and 3-core composite record, including the coefficient of determination (Rsq), the reduction of error (RE), and the coefficient of efficiency (CE) (Fig. 3 b). For the 5-core composite, the coefficient of determination (Rsq) was calculated (1950–1990). Values of RE (CE) above zero indicate some statistical skill in that the reconstructed values over the validation period are better estimates of SST than the mean of the scaling (validation) period^31^. The base period for the scaling was 1961–1990 and comprised two-thirds of the years that the proxy and instrumental SST time series shared in common, with the validation period comprising the remaining one-third. The choice of the scaling and validation period was based on the number of observations in the western Indian Ocean HadSST3 dataset averaged over 10°N-10°S, 50°E-70°E. Well-observed time periods are 1908–1913, 1922–1939 and 1950–1995 (Supplementary Fig. S11).

All time series shown in the manuscript are redisplayed in the supplementary material after removing the linear trends or as first differenced time series. First differenced time series of the coral and local temperature confirm that the corals capture interannual temperature variations (Supplementary Fig. S12). The World War II bias is clearly visible in detrended time series of ERSST4, ERSST3 (Supplementary Fig. S14) and HadSST2 (Supplementary Figs S14 and 15). Detrended versions of HadSST3 and night-time marine air temperature show the same decadal to multidecadal variations as the coral reconstruction (Supplementary Figs S13, S14 and S15). The tight coupling between the Indian Ocean and global mean temperatures is also seen in detrended data (Supplementary Figs S5 and S16).

### Data availability

All methods needed to evaluate the conclusions in the paper are present in the paper and/or the Supplementary Materials. Additional data related to this paper may be requested from the authors. The coral data have been deposited in the World Data Centres for Marine Environmental Sciences and Paleoclimatology, respectively.

## Electronic supplementary material

Supplementary Information
