# Supplementary material for: Indian Ocean corals reveal crucial role of World War II bias for twentieth century warming estimates
Source: Sci Rep. 2017 Oct 31;7:14434. doi: 10.1038/s41598-017-14352-6 (PMC5663710; doi:10.1038/s41598-017-14352-6)
Supplement: Supplementary file 1 — Supplementary Information [file 41598_2017_14352_MOESM1_ESM.pdf]

Supplementary Information:

Indian Ocean corals reveal crucial role of World War II bias for twentieth century warming estimates

Short title: Corals record Indian Ocean warming

Authors

M. Pfeiffer[1]*, J. Zinke[2,3,4,5], W.-C. Dullo[3], D. Garbe-Schönberg[6], M. Latif[7], M. E. Weber[8]

Affiliations

[1*]RWTH Aachen University, Geological Institute, Wüllnerstrasse 2, 52056 Aachen, Germany.

[2]Freie Universitaet Berlin, Section Palaeontology, Malteserstrasse 74-100, 12249 Berlin, Germany.

[3]Curtin University of Technology, Department of Environment and Agriculture, Kent Street, Bentley, WA 6102, Australia.

[4]Australian Institute of Marine Science, PMB 3, Townsville MC, Queensland 4810, Australia.

[5]University of the Witwatersrand, School of Geography, Archaeology & Environmental Studies, Wits 2050, South Africa.

[6]GEOMAR Helmholtz Centre for Ocean Research, Wischhofstrasse 1-3, 24148 Kiel, Germany.

[7]Institute for Geosciences, Kiel University, Ludewig-Meyn-Str. 10, 24118 Kiel, Germany.

[8]Steinmann Institute, University of Bonn, Poppelsdorfer Schloss, 53115 Bonn, Germany

*Corresponding author e-mail: pfeiffer@emr.rwth-aachen.de

Supplementary Methods and Discussion

Coral cores and analytical procedures

Seychelles corals

Core BVB (Beau Vallon Bay), $\delta^{18}O$

A *Porites lutea* colony from Beau Vallon Bay was published in 1997[1], (see Table S1). We refer to this record as core BVB. Beau Vallon Bay is located on the west coast of Mahé (Fig. S1). Core BVB extends from 1846 to 1995 and was measured at 1 mm intervals to obtain a resolution of twelve samples per year[1] (Fig. S2). The age model was developed by assigning the positive $\delta^{18}O$ extremes (=coldest temperatures) in any given year to August 1 (on average the coldest month). Linear interpolation between these anchor points was used to develop a monthly resolved $\delta^{18}O$ time series. The time series can be accessed through the WDC-A Paleoclimatology database (http://www.ngdc.noaa.gov/paleo/data.html). We adjusted the BVB coral to the historical SST products currently available by shifting the positive $\delta^{18}O$ extremes to August 15, as all monthly SST products a centred on day 15 of any given month. For a more detailed description of coral drilling and sampling procedures, the reader is referred to the original publication (see Table S1)[1].

Core NEP (North-East Point), $\delta^{18}O$

Core NEP was drilled in April 1994 from a large *Porites solida* colony growing in a water depth of 9 m off the northeast coast of Mahé (Fig. S1)[2]. The core is 2.03 m long. X-radiographs show clear annual density bands allowing the

development of a precise chronology. The coral has a mean growth rate of approximately 12 mm/year and extends from 1994 to 1840.

The core was sampled for stable isotope analyses at 2 mm intervals, yielding an average of six samples per year. The powdered samples were dissolved in 100% H_3PO_4 at 68°C in an online, automated carbonate preparation device (Kiel device). The resulting CO_2 gas was analysed using a Finnigan Mat 252 mass spectrometer. The standard deviation of multiple samples (N=267) of the international reference NBS-19 was ±0.06 ‰ for $\delta^{18}O$. All data are reported relative to Vienna Peedee Belemnite (VPDB).

The age model of core NEP is based on the strong seasonal cycle in $\delta^{18}O$. This $\delta^{18}O$ chronology is consistent with the annual density bands. We assigned the positive $\delta^{18}O$ extremes to August 15 of every year, which is, on average, the coldest month, and interpolated linearly between those anchor points for all other age assignments. Finally, the $\delta^{18}O$ series was interpolated to six equidistant points per year to obtain a bimonthly time series extending from 1840 to 1994 (Fig. S2).

<u>Chagos corals (Sr/Ca)</u>

The Chagos cores were collected in February 1996 from three massive *Porites* corals growing in the lagoon of Peros Banhos Atoll (Figure S3; Table S1)[3,4]. Core GIM derives from a *Porites solida* colony living in a water depth of 3 m in a channel between Ile Diamant and Grand Ile Mapou, where tidal currents afford good water exchange with the open ocean. Core COI-5 was retrieved from a *Porites lobata* coral living on the lagoon side of Ile du Coin, in very shallow water (1.8 m water depth). Core PIE was taken from a *Porites*

solida colony growing in a water depth of 2.6 m on the lagoon side of Ile Pierre.

X-ray images of core GIM and core PIE show very distinct annual density bands, while the density bands of COI-5 are not very clear. For Sr/Ca analysis, we chose a physical sample spacing of 1 mm, which yielded on average 12-13 samples per year. Samples were taken along the maximum axis of growth. COI-5 and PIE were sampled from August 1949 to February 1996. Core GIM was sampled from August 1879 to February 1996 (Fig. S4). The 1949-1996 Sr/Ca records of all three Chagos corals were compared and validated with local instrumental data in a previous study[3,4]. The 1880-1949 data of core GIM has not been published previously, but analytical procedures and data treatment are the same as in[4].

Sr/Ca ratios were measured at the University of Kiel with a Spectro Ciros CCD SOP Inductively Coupled Plasma Optical Emission Spectrometer (ICP-OES), which simultaneously collects the respective elemental emission signals. The sample solution is prepared by dissolving approximately 0.5 mg of coral powder in 1.00 mL HNO3 70%. The working solution is prepared by serial dilution of the sample solution with HNO3 2% to get a concentration of ca. 8 ppm Ca. Standard solution is prepared by dilution of 1.00 mL of the stock solution (0.52 grams of homogenized coral powder from an in-house standard in 250 mL HNO3 2%) with 2.00 mL HNO3 2%. Sr and Ca lines, which are used for this measurement, are 407 nm and 317 nm respectively. Analytical precision on Sr/Ca determinations is 0.15% RSD or 0.01 mmol/mol (1σ).

The age models of all three Chagos corals were developed based on the seasonal cycle in Sr/Ca[3]. We assigned August 15 to the highest Sr/Ca ratios (=coldest temperatures) measured in any given year, because August is on average the coldest month at Chagos. We then interpolated linearly between these anchor points to obtain age assignments for all other Sr/Ca measurements. In a second step, the Sr/Ca data was interpolated to 12 equidistant points per year to obtain monthly time series. For more details see reference in Table S1.

Composite coral chronology

Table S1 summarizes the coral cores used in the composite chronology and the original publication with further details on the coral sites, methodology and proxy calibration. The Seychelles $\delta^{18}O$ records have been shown to provide excellent records of local SST over the 20th century[1,2]. This is confirmed by $\delta^{18}O_{seawater}$ measurements from the Seychelles, which are generally too small to affect coral $\delta^{18}O$ (shown and discussed in the following section). At Chagos, $\delta^{18}O$ is influenced by $\delta^{18}O_{seawater}$ (precipitation)[3]. Hence, Sr/Ca records have been developed to provide estimates of past SST[3,4]. Sr/Ca is highly correlated with local air temperature and instrumental SST (the latter being relatively sparse/uncertain even in the most recent decades)[4].

The Seychelles (54°E, 5°S) and Chagos (70°E, 5°S) are located in the western and eastern section of the western tropical Indian Ocean (10°N-10°S, 50°E-70°E; Fig. 1, S5), respectively. At both sites, local, satellite-based grid-SST[5] (OI SST) is highly correlated with average western Indian Ocean SST (Fig. S6 and S7). An SST average calculated from the two grid cells including

(i) the Seychelles and (ii) the Chagos shows an excellent match with SST in the western tropical Indian Ocean (Fig. S6 and S7).

To produce the composite coral temperature reconstruction, the BVB core (Seychelles, $\delta^{18}O$) was resampled to bimonthly resolution. We then computed the arithmetic mean of both cores (Fig S2). The monthly resolved Chagos Sr/Ca records were also averaged, and then resampled to bimonthly resolution (Fig. S4). The final coral composite is the arithmetic mean of the Chagos and Seychelles chronologies (Fig. 2).

Seychelles: δ^{18}O seawater

At the Seychelles, $\delta^{18}O_{seawater}$ was measured at biweekly intervals between August 20, 2001 and December 2, 2002, i.e. over a 1.5 year period (samples from May 2002 were lost during shipping). Depending on weather conditions, samples were taken on the Island of St. Anne (located in the NE of Mahé) and/or at the Northeast Point on Mahé, the main island of the Seychelles. Water samples were filled into 100ml crimp sealed bottles and 0.2ml of a saturated $HgCl_2$ solution was added to prevent biological activity. The oxygen isotopes were analysed at the Leibniz Institute in Kiel (Germany) following the method described in[6]. The measurement precision for $\delta^{18}O_{seawater}$ is 0.06 % (1 σ). A total of 38 samples was analysed. The $\delta^{18}O_{seawater}$ data is shown in Fig. S8. $\delta^{18}O_{seawater}$ has a mean of 0.59 per mill and a standard deviation of +/- 0.06 permill (1σ), i.e. the standard deviation does not exceed the analytical precision. The time series of $\delta^{18}O_{seawater}$ shows random fluctuations around the mean value, but no apparent seasonality. 10 samples lie outside the 1σ range, and 2 samples are outside 2σ. (For N = 38, we would expect 13 values

outside 1σ, and 1.8 values outside 2σ by chance). We therefore conclude that seasonal changes in $\delta^{18}O_{seawater}$ are too small to affect coral $\delta^{18}O$ measurements, which have the same analytical precision as $\delta^{18}O_{seawater}$.

To assess potential longer-term fluctuations in $\delta^{18}O_{seawater}$ we extracted $\delta^{18}O_{seawater}$ data for the region 0-7.5°S, 50°-65°E from the Global Seawater Oxygen-18 Database[7]. The database contains $\delta^{18}O_{seawater}$ data collected in January 2006 along a transect from 59°-62°E, 1°-7.5°S (n=13)[8]. Some additional data points (n=5) are reported from 54°-54.5°E, 0°-1°S [9]. For this data, the exact time of collection is uncertain, but all water samples are from the early 1990s[9]. For both datasets, an analytical precision of 0.1 permil is reported[8,9]. The $\delta^{18}O_{seawater}$ measurements have a mean value of 0.54 per mil and a standard deviation of 0.09 permil. This indicates that temporal and spatial variations of $\delta^{18}O_{seawater}$ are smaller than the measurement precision of $\delta^{18}O_{seawater}$ (and coral $\delta^{18}O$) in the western Indian Ocean. This has been confirmed in a core-top calibration study using paired coral $\delta^{18}O$ and Sr/Ca measurements from a Seychelles coral[10]. Paired $\delta^{18}O$ and Sr/Ca measurements from Holocene Seychelles corals also do not show a significant contribution of $\delta^{18}O_{seawater}$[10].

Air temperature data

Monthly air temperature records are available from Diego Garcia (Chagos, WMO station code: 61967) and the Seychelles (WMO station code: 63980). Both stations have several data gaps. At each site we have computed annual averages for years with at least 10 months of data (Fig. S9). For years with data from each site, we calculated the arithmetic mean. In several years we

have data from only one station (Fig. S9). The Chagos air temperature record extends back until 1955 and ends in 1993.

HadSST3: uncertainties

Annual average sea-surface temperatures and their uncertainties were calculated using the HadSST3 methodology[11,12]. Briefly, there are three sources of uncertainty: uncertainty in the adjustments for systematic errors in the data associated with changes in measurements method over time, uncertainties arising from uncorrelated or partially correlated measurement errors and uncertainties due to incomplete spatial coverage of the area of interest. Uncertainties in the adjustments for systematic errors are assessed by varying uncertain parameters used in the adjustment scheme within their likely ranges[12]. Uncertainties associated with measurement errors for the study region (10N-10S 50E-70E) were calculated in each grid box as in HadSST3[11]. The uncertainties on the individual monthly grid box averages were combined as if they were uncorrelated and then multiplied by a factor of 1.604 to get an estimate of the combined uncorrelated and partially correlated uncertainty. This factor was calculated for the study region in the same way in which global and hemispheric correction factors were calculated in HadSST3. The monthly area-averages over the study region were then averaged to get an estimate of the annual area-average and the uncertainties were combined using the same effective number of months (2.25) as were used in the global average in HadSST3. The uncertainty due to limited coverage was estimated by subsampling a globally complete SST analysis[12]. Figure S10 shows the relative contributions of each type of uncertainty to the total.

HadSST3: number of observations

SST observations made by voluntary observing ships are temporally and spatially discontinuous. Fig. S11 shows the number of observations used in the HadSST3 dataset per 5°x5° grid in the 50°E-70°S and 10°N-10°S region (this data can be accessed at http://www.metoffice.gov.uk/hadobs/hadsst3/data/download.html). Most observations are in the grid boxes located north of the equator. Data density is highest between 1908 - 1913, 1922 - 1939 and from 1950 to present.

Effect of linear trend on correlations

Since linear trends may inflate the correlation between two time series, all time series shown in the manuscript are redisplayed without their linear trends (Fig. S5, Fig. S12-Fig. S16). We have subtracted the linear trends estimated by OLS regression. As the 1955-1995 period covered by the local temperature record is dominated by the regime shift in 1975, rather than a linear trend, the coral and instrumental data shown in Fig. 2 c and d are redisplayed as first differenced time series/data in Fig. S12.

References

S1. Charles, C. D., Hunter, D. E., Fairbanks, R. G. Interaction between the ENSO and the Asian Monsoon in a coral record of tropical climate. *Science* **277,** 925-928 (1997).

S2. Pfeiffer, M., Dullo, W.-C. Monsoon-induced cooling of the western equatorial Indian Ocean as recorded in coral oxygen isotope records from the

Seychelles covering the period of 1840–1994 AD. *Quat. Sci. Rev.* **25,** 993–1009 (2006).

S3. Pfeiffer, M., Timm, O., Dullo, W.-C., Garbe-Schoenberg, D. Paired coral Sr/Ca and δ^{18}O records from the Chagos Archipelago: Late twentieth century warming affects rainfall variability in the tropical Indian Ocean. *Geology* **34,** 1069-1072 (2006).

S4. Pfeiffer, M., Dullo, W.-C., Zinke, J., Garbe-Schönberg, D. Three monthly coral Sr/Ca records from the Chagos Archipelago covering the period of 1950–1995 A.D.: reproducibility and implications for quantitative reconstructions of sea surface temperature variations. *Int. J. Earth Sci.* **98,** 53–66, doi: 10.1007/s00531-008-0326-z (2009).

S5. Reynolds, R. W., Rayner, N.A., Smith, T.M., Stokes, D. C., Wang, W. An improved in situ and satellite SST analysis for climate. *J. Clim.* **15,** 1609-1625 (2002).

S6. Bauch, D., Erlenkeuser, H., Andersen, N. Water mass processes on Arctic shelves as revealed from delta18O of H2O, *Global Planet. Change* **48,** 165-174 (2005).

S7. Schmidt, G.A., Bigg G.R., Rohling, E.J. "Global Seawater Oxygen-18 Database - v1.21" https://data.giss.nasa.gov/o18data/ (1999).

S8. Srivastava, R., Ramesh, R., Prakash, S., Anilkumar, N. Sudhakar, M. Spatial variation of oxygen isotopes and salinity in the Southern Indian Ocean, *Geophys. Res. Lett.* **34,** doi:10.1029/2007GL031790 (2007).

S9. Delaygue, G., Bard E., Rollion C., Jouzel J., Stievenard M., Duplessy J.-C. and Ganssen G., Surface Water Measurements of Salinity and Oxygen-18 in the Northern Indian Ocean, *J. Geophys. Res.* **106,** 4565 (2001).

S10. Zinke, J., Pfeiffer, M., Park, W., Schneider, B., Reuning, L., Dullo, W.-Chr., Camoin, G.F., Mangini, A., Schroeder-Ritzrau, A., Garbe-Schönberg, D. and Davies, G.R., Seychelles coral record of changes in sea surface temperature bimodality in the western Indian Ocean from the Mid-Holocene to the present, *Climate Dynamics* 689-708 (2014).

S11. Kennedy J.J., Rayner, N.A., Smith, R.O., Saunby, M. and Parker, D.E. Reassessing biases and other uncertainties in sea-surface temperature observations since 1850 part 1: measurement and sampling errors. *J. Geophys. Res.* **116,** D14103, doi:10.1029/2010JD015218 (2011).

S12. Kennedy J.J., Rayner, N.A., Smith, R.O., Saunby, M. and Parker, D.E. Reassessing biases and other uncertainties in sea-surface temperature observations since 1850 part 2: biases and homogenisation. *J. Geophys. Res*. **116,** D14104, doi:10.1029/2010JD015220 (2011).

S13. Hansen, J. Ruedy, R., Sato, M., Lo, K. Global surface temperature change. *Rev. Geophys.* **48,** RG4004, doi:10.1029/2010RG000345 (2010).

S14. Juillet-Leclerc, A., Schmidt, G. A calibration of the oxygen isotope paleothermometer of coral aragonite from *Porites*. *Geophys. Res. Lett*. **28,** 4135-4138 (2001).

S15. Corrège, T. Sea surface temperature and salinity reconstructions from coral geochemical tracers. *Palaeogeography, Palaeoclimatology, Palaeoecology* **232,** 408-428 (2006).

S16. Huang, B., Banon, V. F., Freeman, E., Lawrimore, J., Liu, W., Peterson, T. C., Smith, T. M., Thorne, P. W., Woodruff, S.D., Zhang, H.-M. Extended Reconstructed Sea Surface Temperature Version 4 (ERSST.v4). Part I:

Upgrades and Intercomparisons. *J. Clim.* **28,** 911-930, doi: 10.1175/JCLI-D-14-00006.1 (2015).

S17. Smith, T. M., Reynolds, R. W., Peterson, T. C., Lawrimore, J. Improvements to NOAA's historical merged land–ocean surface temperature analysis (1880–2006). *J. Clim.* **21,** 2283–2296 (2008).

S18. Rayner, N. A., Brohan, P., Parker, D. E., Folland,C. K., Kennedy, J. J., Vanicek, M., Ansell, T. J., Tett, S. F. B. Improved analyses of changes and uncertainties in sea surface temperature measured in situ since the mid-nineteenth century: The HadSST2 data set. *J. Clim.* **19,** 446–469 (2006).

S19. Parker, D. E., Folland, C. K., Jackson M. Marine surface temperature: Observed variations and data requirements, Clim. Chang. 31, 559–600 (1995).

S20. Kent, E. C., Rayner, N. A., Berry, D. I., Saunby, M., Moat, B. I., Kennedy, J. J., Parker, D. E. Global analysis of night marine air temperature and its uncertainty since 1880: The HadNMAT2 data set. *J. Geophys. Res. Atmos.* **118,** 1281–1298, doi:10.1002/jgrd.50152 (2013).

S21. Morice, C. P., Kennedy, J. J., Rayner, N. A., Jones, P.D. Quantifying uncertainties in global and regional temperature change using an ensemble of observational estimates: The HadCRUT4 dataset. *J. Geophys. Res.* **117,** D08101, doi:10.1029/2011JD017187 (2012).

Supplementary Tables 1-6

Table S1 Coral cores included in the composite temperature reconstruction.

Core	location	proxy	time period	reference
BVB	Mahé, Seychelles	$\delta^{18}O$	1847-1995	Charles et al., 1997(S1)
NEP	Mahé, Seychhelles	$\delta^{18}O$	1840-1994	Pfeiffer & Dullo, 2006 (S2)
GIM	Peros Banhos, Chagos	Sr/Ca	1880-1995	Pfeiffer et al., 2009 (S4)
COI-5	Peros Banhos, Chagos	Sr/Ca	1950-1995	Pfeiffer et al., 2009 (S4)
PIE	Peros Banhos, Chagos	Sr/Ca	1950-1995	Pfeiffer et al., 2009 (S4)

Table S2 Validation of coral temperature estimates. Ordinary least squares (OLS) regression of the coral temperature reconstruction vs. the air temperature/OI SST composite from Chagos and the Seychelles (1955-1995). All correlations are significant at the 1% level assuming 38 degrees of freedom (n=40).

1995-1993	R	sigma	slope (95% CI)
5 cores	0.82	0.22	0.95 (0.72/1.19)
3 cores	0.65	0.24	0.64 (0.38/0.90)
2 cores	0.53	0.30	0.59 (0.27/0.91)

Table S3 Validation of coral temperature estimates: first differenced time series. Ordinary least squares (OLS) regression of the coral temperature reconstruction vs. the air temperature/OI SST composite from Chagos and the Seychelles (first differenced time series; 1955-1995). All correlations are significant at the 1% level assuming 37 degrees of freedom (n=39).

1995-1993	R	sigma	slope (95% CI)
5 cores	0.78	0.23	0.79 (0.58/1.00)
3 cores	0.77	0.23	0.78 (0.56/0.99)
2 cores	0.65	0.28	0.67 (0.41/0.94)

Table S4 Standard deviation: Coral and local temperature data. Standard deviation of the air temperature/OI SST composite and the coral temperature composites (annual means and first differenced time series). Time period: 1955-1995.

	St. dev. (°C), annual means	St. dev. (°C), 1. differenced series
air/OI temperature	0.32	0.35
5 core composite	0.37	0.35
3 core composite	0.32	0.34
2 core composite	0.36	0.36

Table S5 Standard deviation: Coral and western Indian Ocean temperature. Standard deviation of historical temperature data averaged over 50°-70°E, 10°S-10°N and coral temperature composites. Time period: 1961-1990.

	St.dev. (°C), annual means
ERSST3	0.20
ERSST4	0.22
HadSST2	0.24
HadSST3	0.21
HadMAT1	0.25
HadNMAT2	0.22
5 cores	0.36
3 cores	0.31
2 cores	0.33

Table S6 Indian Ocean warming rates. Warming rates (°C/decade) of the western tropical Indian Ocean **(**50°E,-70°E, 10°S-10°N) estimated usind OLS regression. The corals have been adjusted to the standard deviation of HadSST3 (base period 1961-1990; see manuscript text).

	**1900**	**95% CI**		**1950**	**95% CI**	
Coral adjusted	0.065	0.057	0.082	0.075	0.026	0.124
ERSST4	0.083	0.067	0.098	0.145	0.105	0.185
ERSST3	0.081	0.067	0.095	0.137	0.095	0.179
HadSST3	0.060	0.045	0.074	0.051	0.007	0.095
HadSST2	0.060	0.044	0.076	0.146	0.099	0.192

Supplementary Figures 1-16

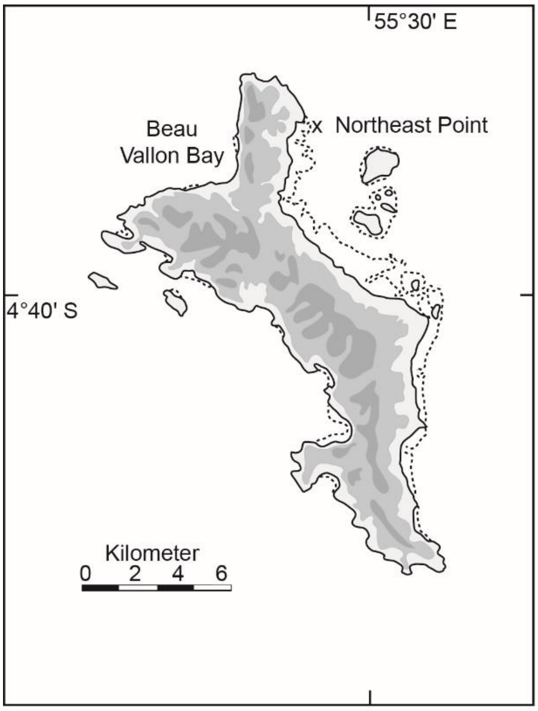

Fig. S1. Map of Mahé Island, Seychelles. Stipled line delineates the reef front. Sampling location of core NEP indicated by x. Core BVB is from Beau Vallon Bay. See Table S1 for further details. Map created in Adobe Illustrator CS 2.

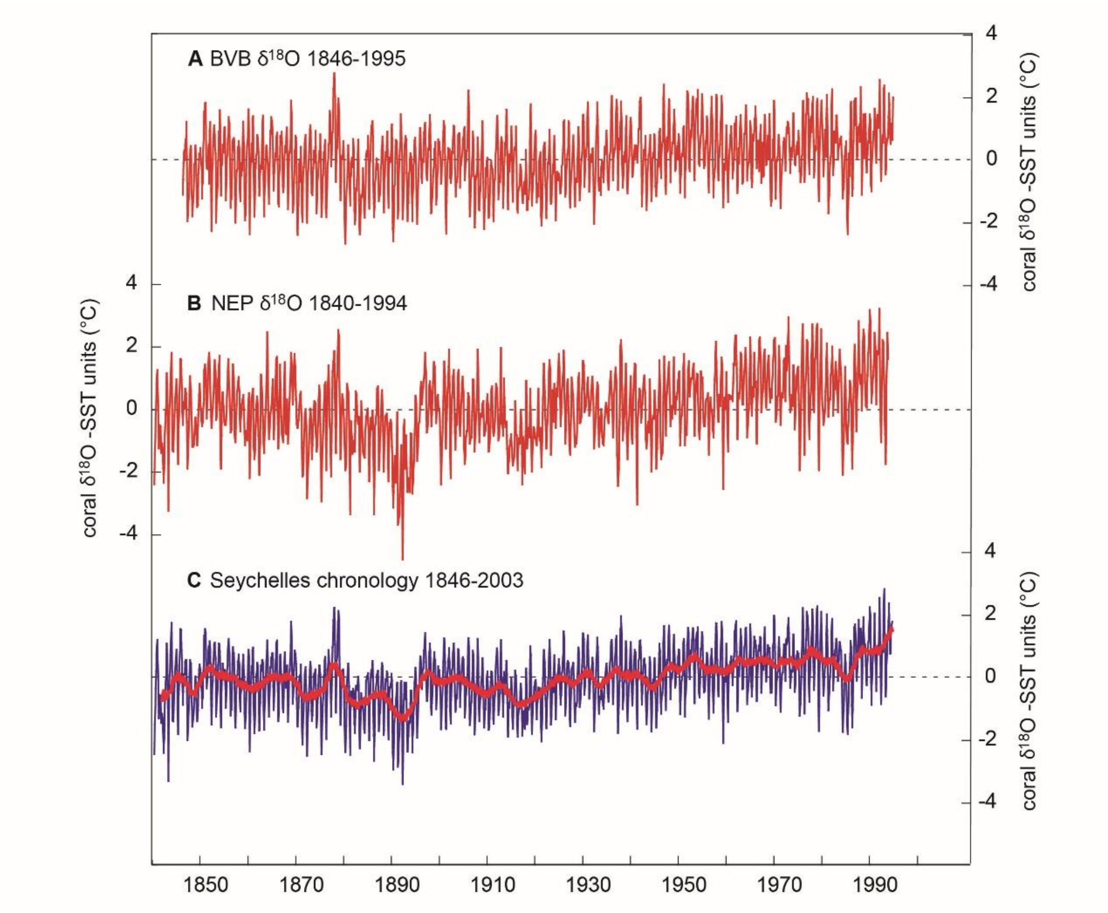

Fig. S2. Seychelles chronology. The Seychelles chronology comprises two coral $\delta^{18}O$ time series from the island of Mahé: **a)** A monthly resolved $\delta^{18}O$ record that extends from 1846-1995[1], **b)** A bimonthly resolved $\delta^{18}O$ record that extends from 1840-1994[2]. All $\delta^{18}O$ series have been centered by removing the mean of the 1961-1990 period and converted to temperature units using the mean coral $\delta^{18}O$-temperature relationship of -0.2 per mill[14] per 1°C. **d)** The composite chronology (blue thin line: bimonthly data, thick red line 31 point moving average) is the arithmetic mean of two coral $\delta^{18}O$ records.

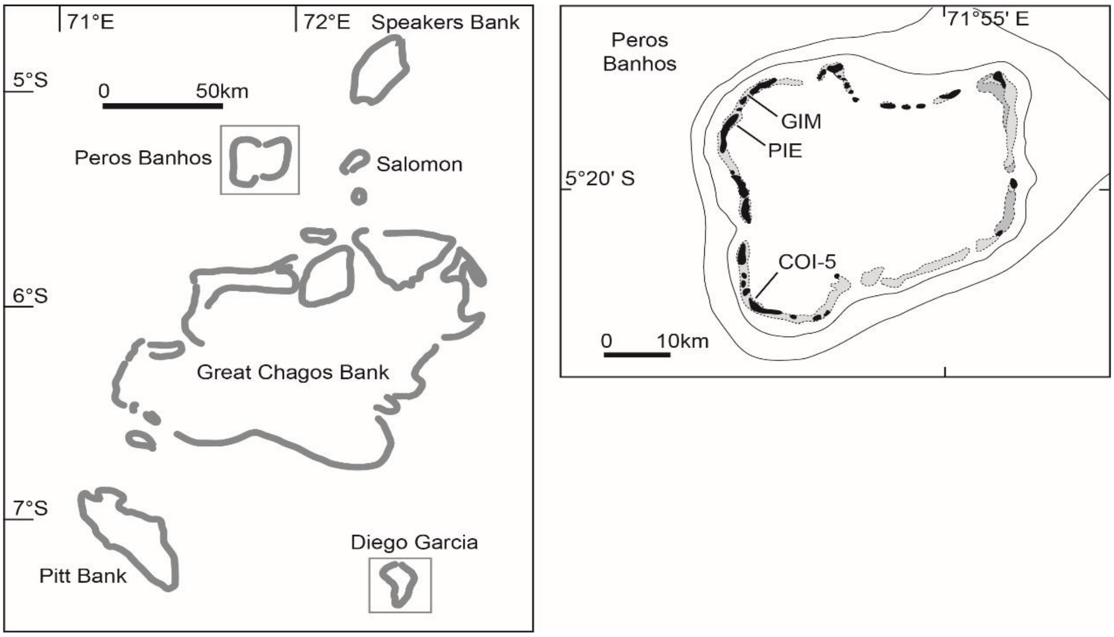

Fig. S3. The Chagos Archipelago. Grey squares mark location of Peros Banhos, where the cores have been drilled, and Diego Garcia, where air temperature data is available (WMO Station ID 6196700). See Table S1 for further details. Map created in Adobe Illustrator CS 2.

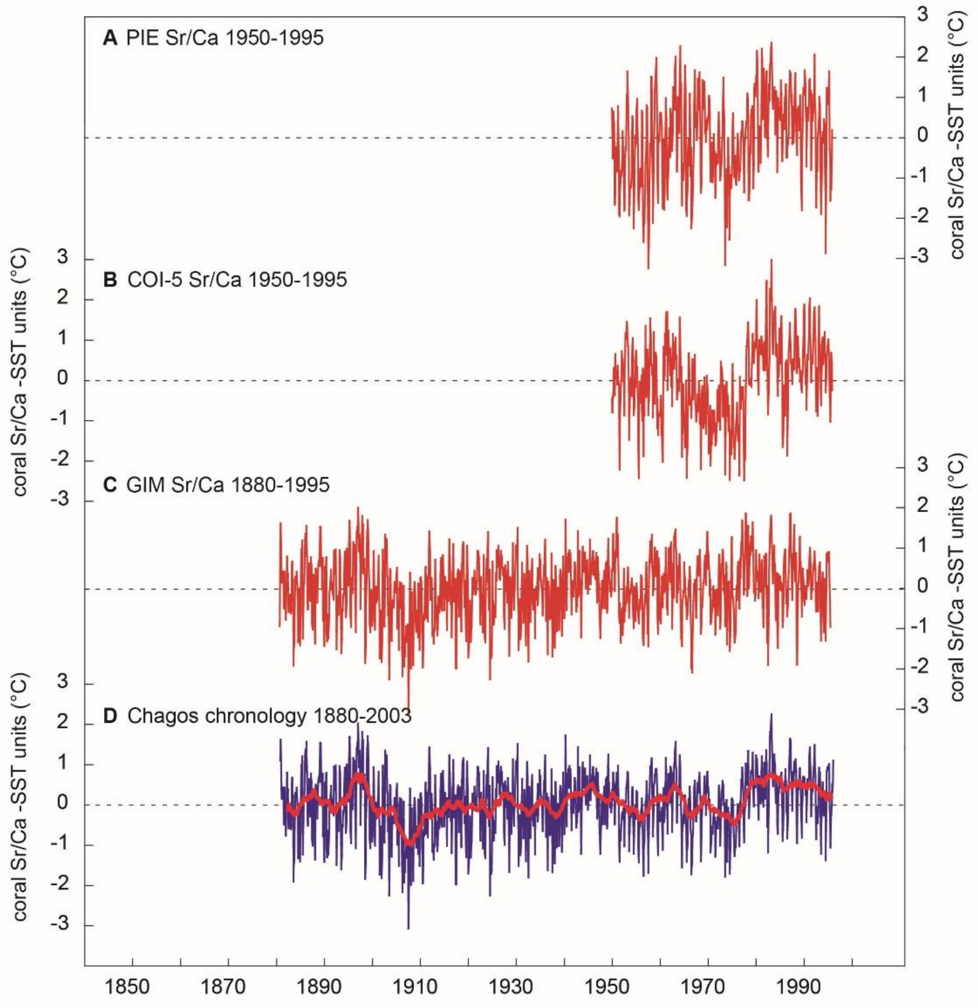

Fig. S4. The Chagos chronology. The Chagos chronology comprises three monthly resolved coral Sr/Ca time series drilled at Peros Banhos Atoll, in the north of the Chagos Archipelago[3,4]. **a)** and **b)** two shorter cores extend from 1950-1995[4], **C.** one long core extends from 1880-1995 (only the top 50 years of this record have been published previously[3,4]). All Sr/Ca series have been centered by removing the mean of the 1961-1990 period and converted to temperature units using the mean coral Sr/Ca-temperature relationship[15] of -0.06 mmol/mol per 1°C. **d)** The composite chronology (blue thin line: monthly data, thick red line 31 point moving average) is the arithmetic mean of the three records. All cores end in 1995.

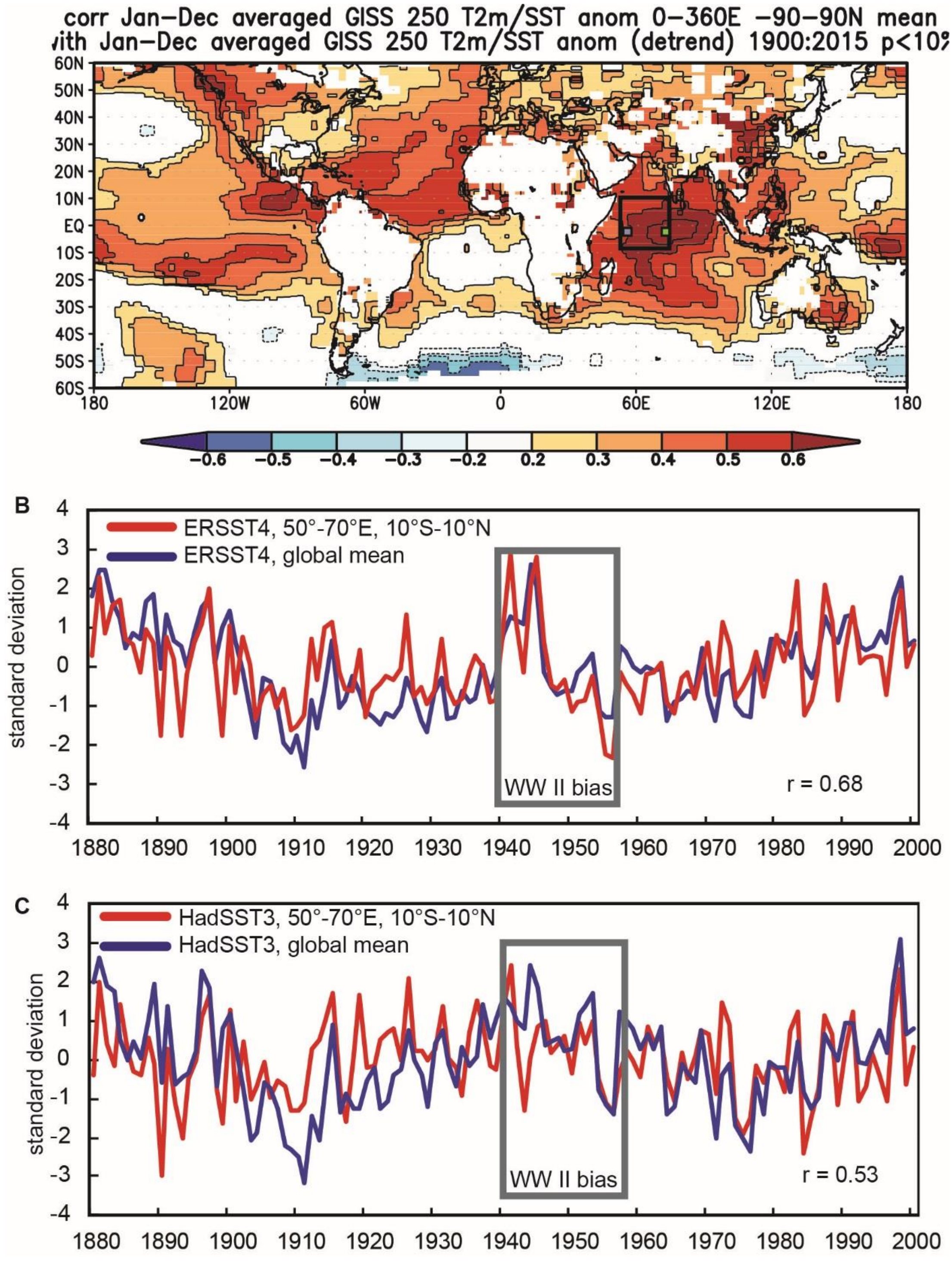

Fig. S5. Historical Indian Ocean and global mean temperature. Historical Indian Ocean and global mean temperature shown in Fig. 1 after removing the linear trends from all datasets. **a)** Field correlation of the global mean temperature record with gridded temperature data (GISSTEMP 250 km

resolution, blended surface air temperature and SST[13]). Note the high correlation in the tropical Indian Ocean. The black rectangle indicates the target region of our study: the western tropical Indian Ocean (10°N-10°S, 50°E-70°E). The small rectangles indicate the location of the Seychelles (green) and the Chagos Archipelago (blue). Correlations not significant at the 10% level are masked out. Field correlation computed at http://climexp.knmi.nl and edited in Adobe Illustrator. **b)** Instrumental SST of the western tropical Indian Ocean extracted from the ERSST4 product[16] (thin blue line: annual means; thick blue line 11 point moving averages) compared with global mean ERSST4 data (thin red line: annual means; thick red line: 11 point moving averages). **c)** Same as **b)** but for HadSST3[11,12]. In each case, western Indian Ocean and global mean SST are highly correlated (annual means: r = 0.53 and 0.63, respectively, significant at the 1 % level assuming 118 degrees of freedom, N=120). The temporal pattern of the warming depends on the SST product. Discrepancies are largest during and after the World War II period (1940-1960, grey rectangle).

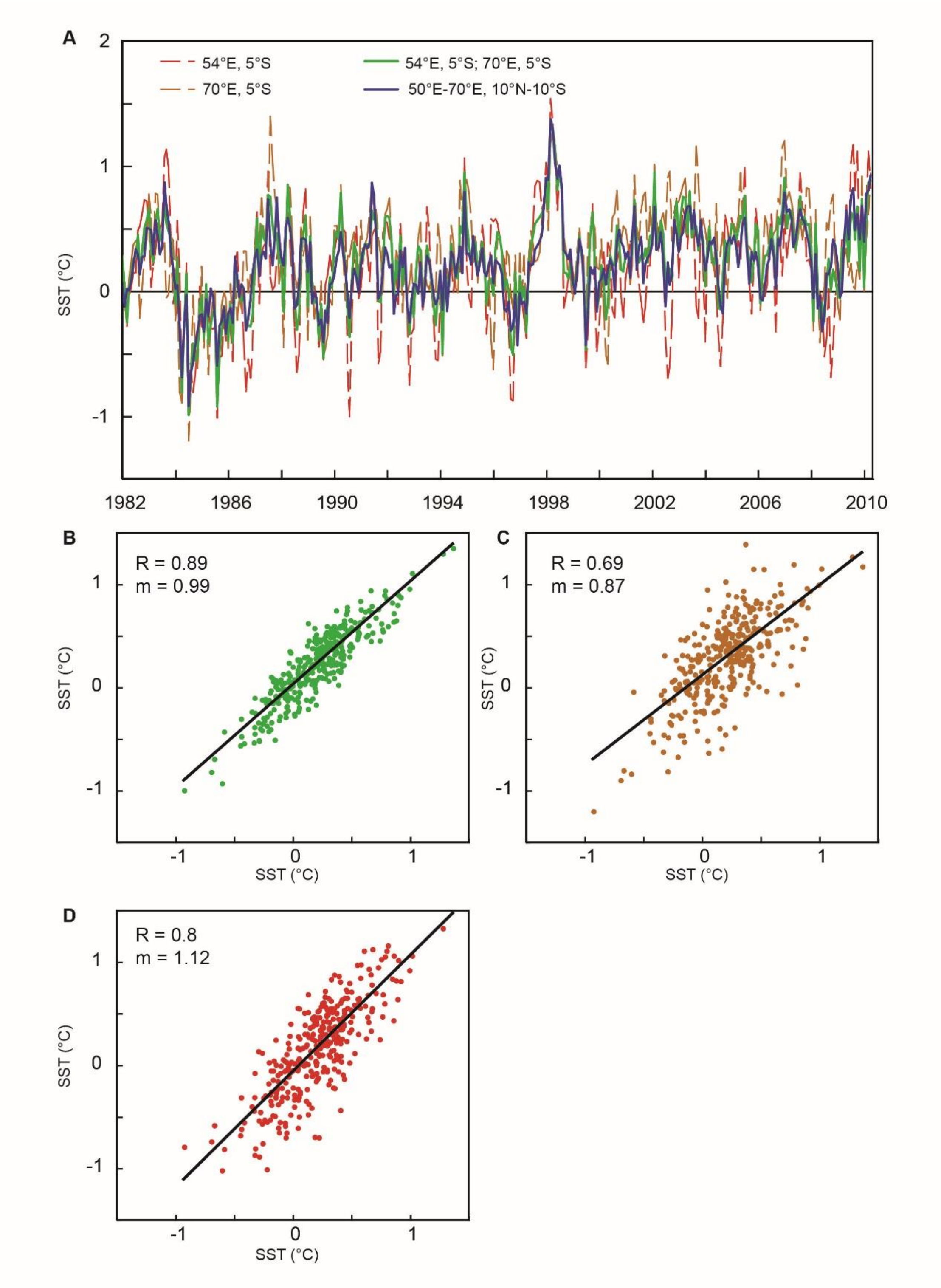

Fig S6. Satellite SST: The Seychelles, Chagos and the western tropical Indian Ocean. a) Satellite SST[5] (monthly anomalies). Dashed red line: Seychelles SST (54°E, 5°S), dashed yellow line: Chagos SST (70°E, 5°S), green solid line: average Seychelles and Chagos SST (54°E, 5°S and 70°E,

5°S). Blue solid line: regional SST in the western Indian Ocean (10°N-10°S, 50-70°E). **b)** Linear regression of the Seychelles and Chagos SST average vs. SST in the western Indian Ocean. **c)** Same as b but for Seychelles SST only. **d)** Same as b but for Chagos SST. R: correlation coefficient, m: slope of the linear regression.

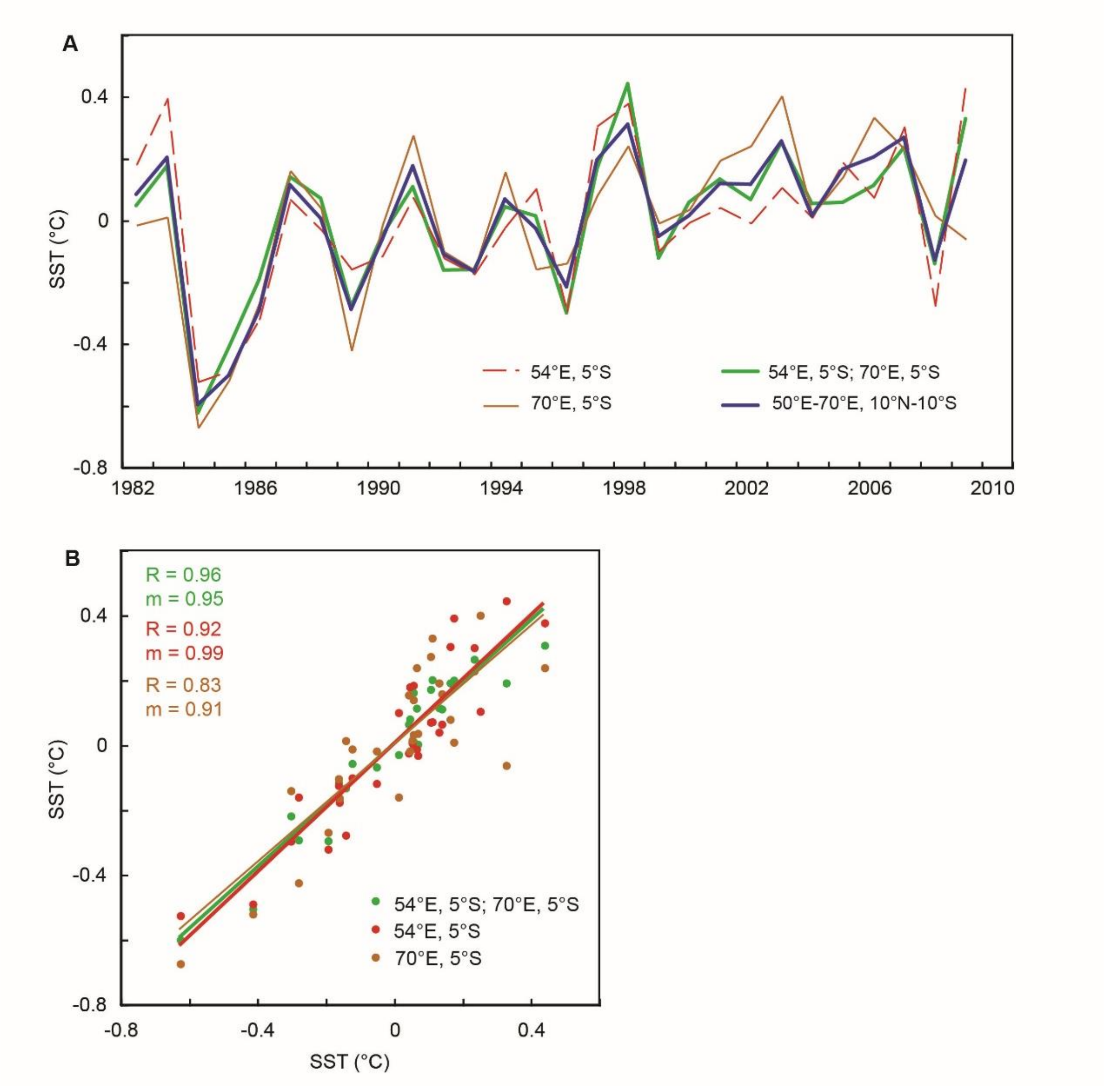

Fig S7. Satellite SST: The Seychelles, Chagos and the western tropical Indian Ocean: annual mean data. a) Satellite SST[5] (annual means). Dashed red line: Seychelles SST (54°E, 5°S), dashed yellow line: Chagos SST (70°E, 5°S), green solid line: average Seychelles and Chagos SST (54°E, 5°S and 70°E, 5°S). Blue solid line: regional SST in the Western Indian Ocean (10°N-

10°S, 50-70°E). **b)** Linear regression of Seychelles and Chagos SST average (green dots), Seychelles SST (red dots) and Chagos SST (yellow dots) vs. SST in the Western Indian Ocean. R: correlation coefficient, m: slope of the linear regression.

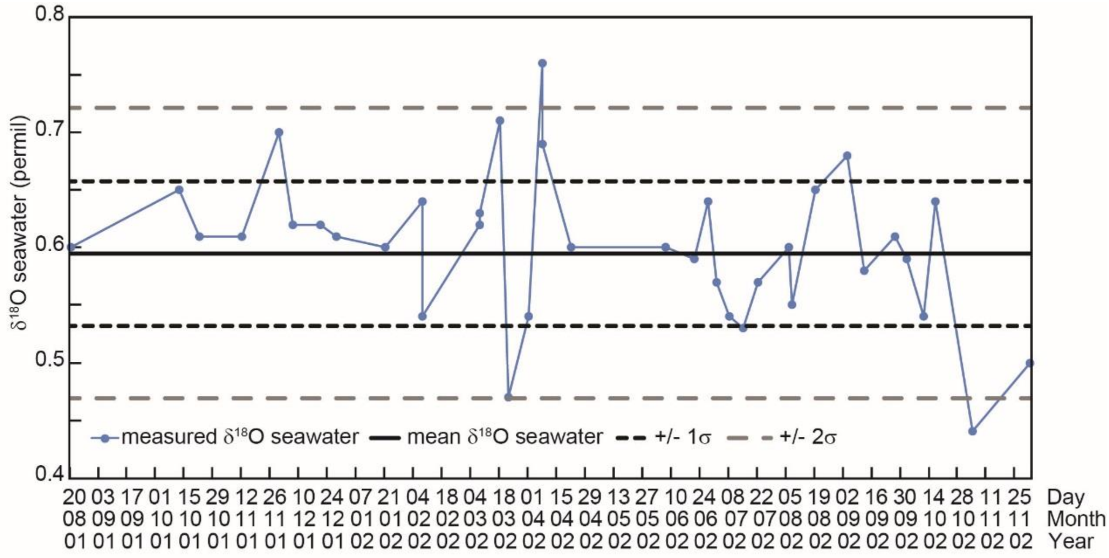

Fig. S8. Seawater $\delta^{18}O$ measured at the Seychelles. Seychelles $\delta^{18}O_{seawater}$ data (blue line and dots), measured at biweekly intervals between August 20, 2001 and December 2, 2002, i.e. over a 1.5 year period (N=38; samples from May 2002 were lost during shipping). $\delta^{18}O_{seawater}$ has a mean of 0.59 per mill (thick black line). Black (grey) dashed line: 1σ (2σ) of analytical uncertainty (+/- 0.06 per mill). See Supplementary Methods and Discussion.

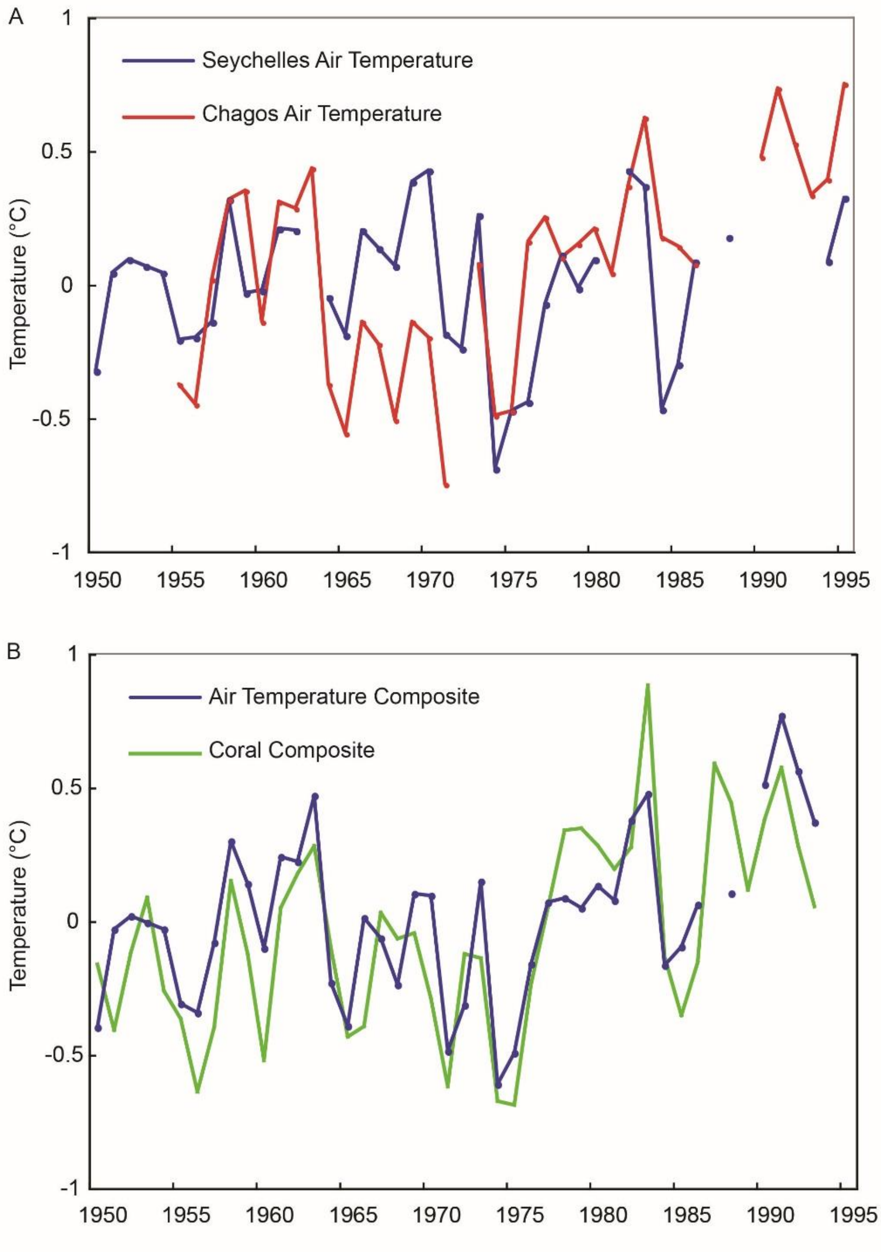

Fig. S9. Air temperature at the Seychelles and Chagos. a) Air temperature data from the Seychelles (blue line) and Chagos (red line). **b)** Composite air temperature record and composite coral chronology. Time series are centered by removing the mean of the 1961-1990 period. The corals have been converted to temperature using the mean proxy-SST relationships ($\delta^{18}O$: -0.2 permil per 1°C[14] and Sr/Ca: -0.06 mmol/mol per 1°C[15]).

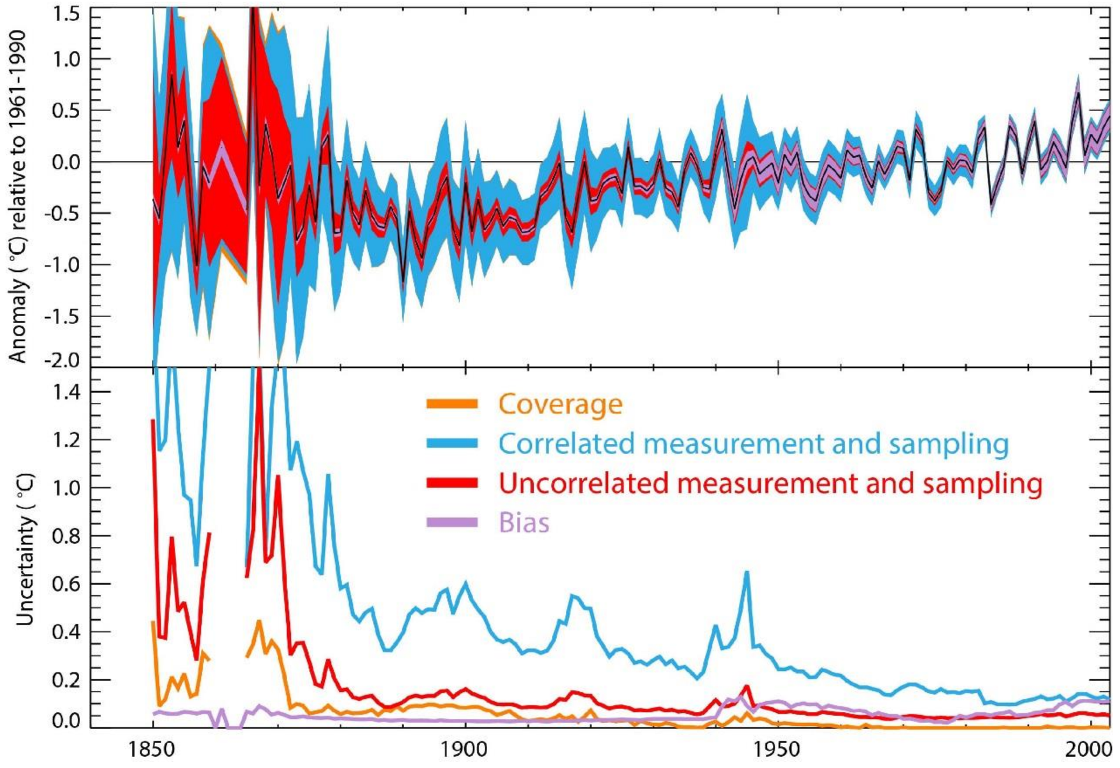

Fig. S10. Western Indian Ocean HadSST3: uncertainties. Annual average sea-surface temperature anomalies (upper panel, relative to the 1961-1990 average) for the western Indian Ocean (10°N-10°S, 50-70°E). The black line shows the best estimate and the coloured areas shows the additive effect of the various contributions to the total 2-sigma uncertainty. The contributions are from uncertainty in the bias adjustments (lilac), uncorrelated measurement and sampling uncertainty (red), partially correlated measurement and sampling uncertainty (blue) and the coverage uncertainty (orange). The lower panel shows the individual contributions as their relative magnitudes are difficult to assess from the top panel where they are combined in quadrature.

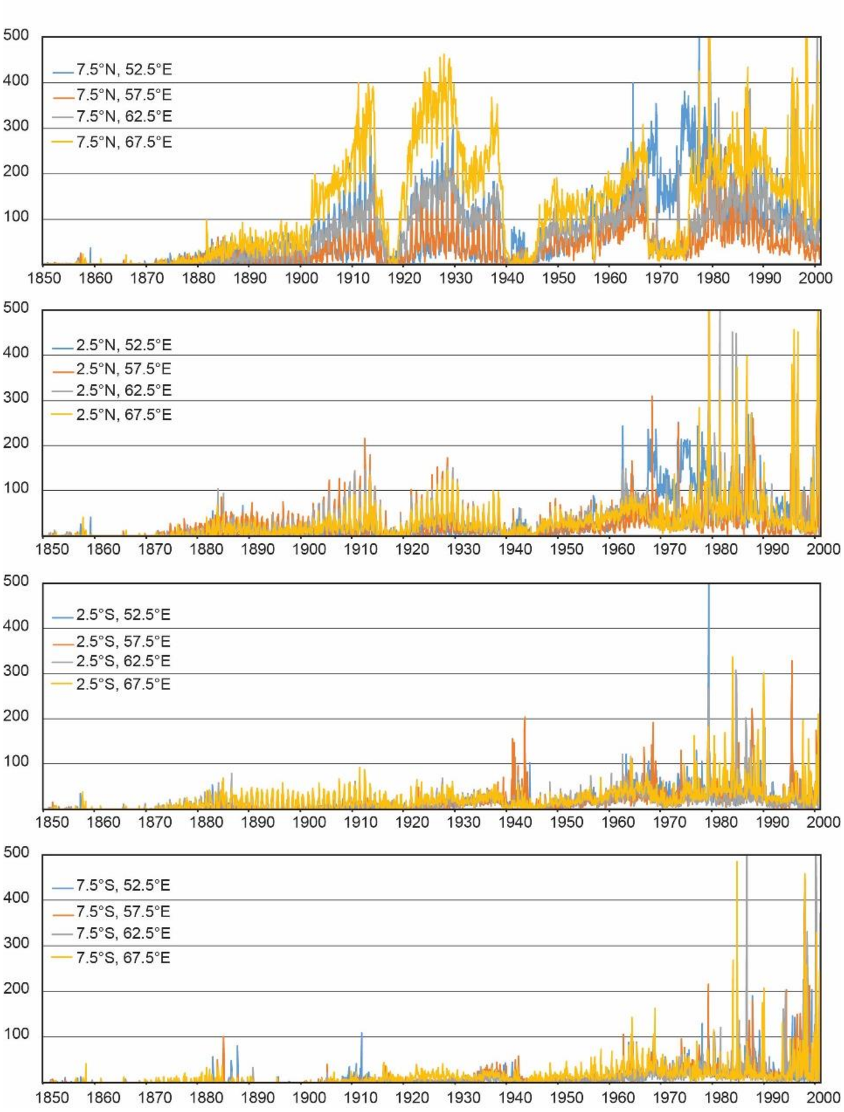

Fig. S11. Western Indian Ocean HadSST3: number of observations. Number of observations per 5°x5° grid contained in HadSST3[11,12] in the region 50°-70°E, 10°N-10°S. Note: most observations are from the grid centered at 7.5°N, 67.5°E. The time periods between 1908-1913, 1923-1939 and 1950-present are well observed.

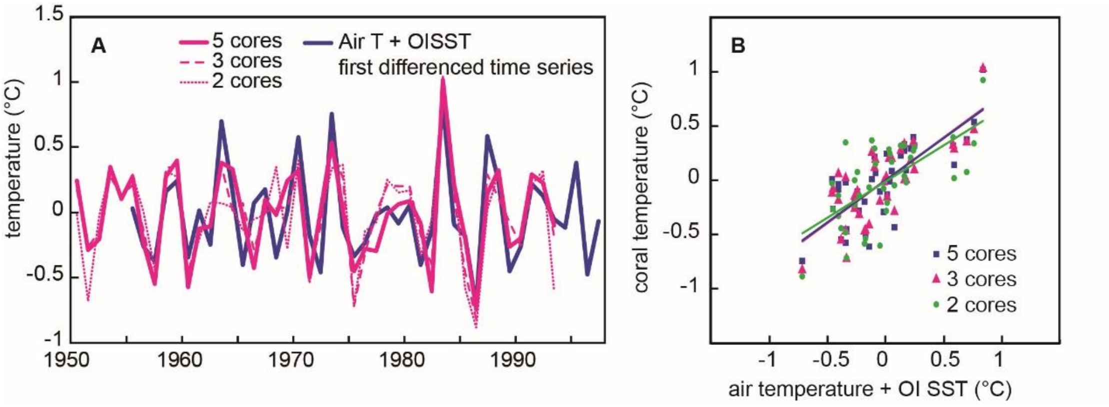

Fig. S12. Coral temperature reconstruction: validation. a, b) First differenced time series of the coral and local temperature data shown in Fig. 2 **c)** and **d)** of the main manuscript. **a)** Comparison of the coral derived temperatures (5, 3 and 2 core averages) with a composite record computed from local air temperature (Mahe, Seychelles, and Diego Garcia, Chagos; see Fig. S7) and local OI SST (1°x1° grid, available since 1982[5]). **b)** Scatter plot of first differenced coral derived temperatures (5, 3 and 2 core composites) vs. local air temperature/OI SST. Correlation coefficients are reported in Table S3.

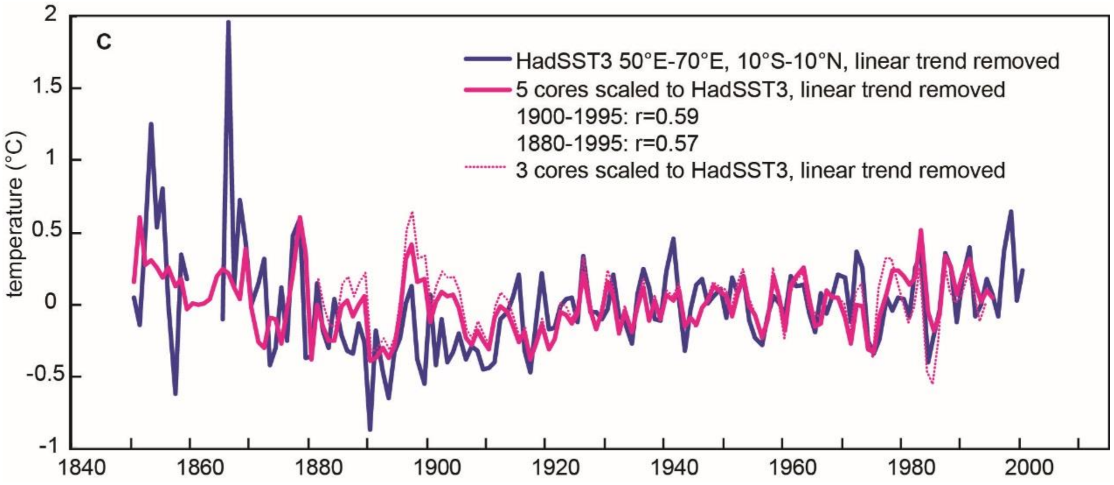

Fig. S13. Western Indian Ocean coral temperature reconstruction. WIO coral temperature reconstruction (magenta) and HadSST3[11,12] 50°E-70°E, 10°N-10°S (blue) shown in Fig. 3 of the manuscript after removing the linear trends (annual means: r = 0.59 for 1900-1995, significant at the 1 % level assuming 93 degrees of freedom, N=95).

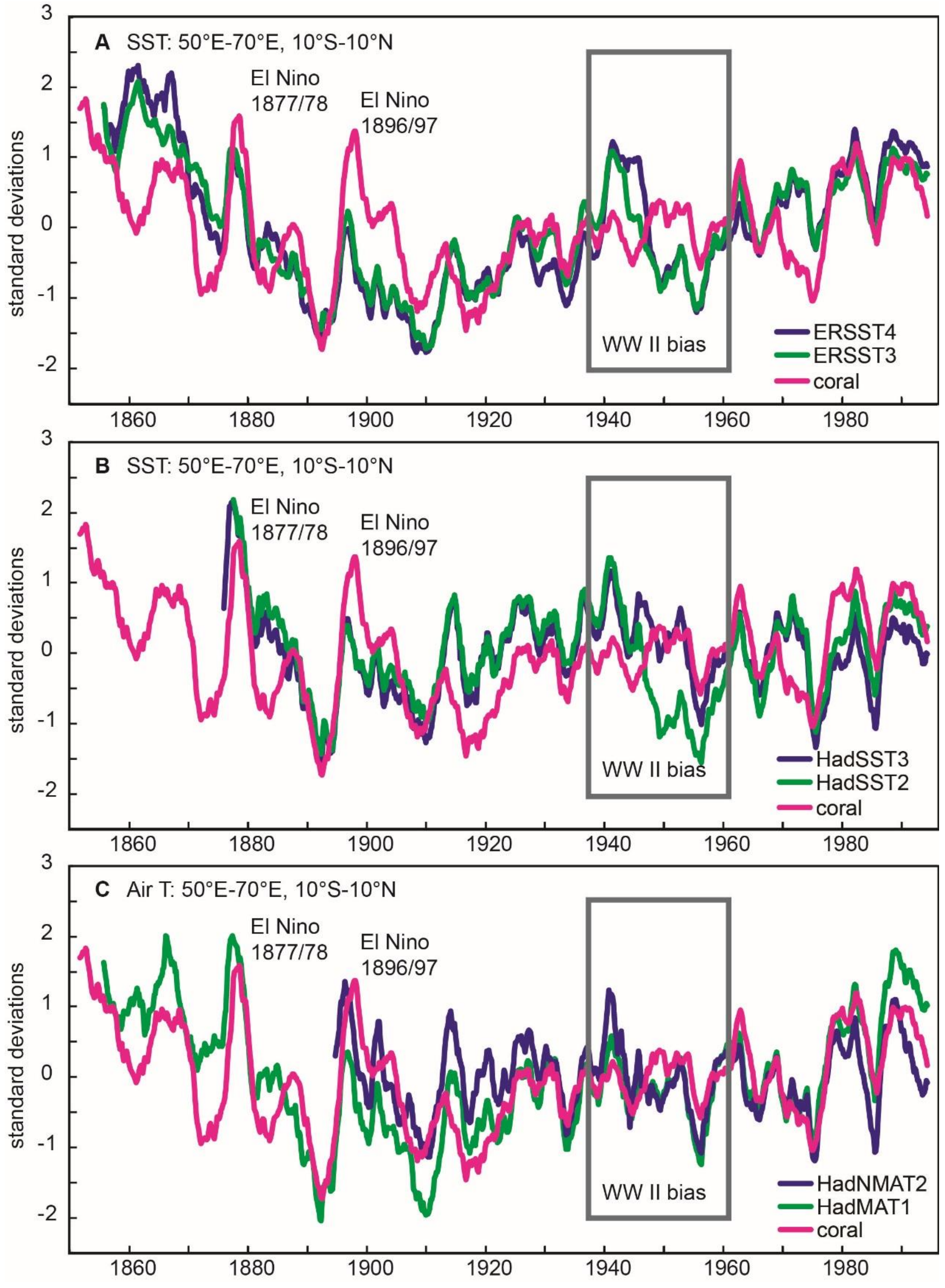

Fig. S14. Western Indian Ocean coral and historical temperatures: WW II bias. Western Indian Ocean (WIO) coral and historical temperature data shown in Fig. 4 of the manuscript after removing the linear trends. **a)** WIO coral temperature reconstruction (magenta) compared with WIO ERSST4[16]

(green) and ERSST3[17] (blue). **b)** Same as **a)** using HadSST2[18] and HadSST3[11,12]. **c)** Same as **a)** using night-time marine air temperature (HadMAT1[19] and HadNMAT2[20]). All time series are 21 point moving averages of bimonthly anomalies, and have been normalized to their standard deviation over the 1900-1995 period. Note differences during and after World War II depending on the SST product (black bar: time period of World War II bias). WIO coral indicates large amplitude warm temperature anomalies during the late 19th century El Niño events of 1877/78 and 1896/97.

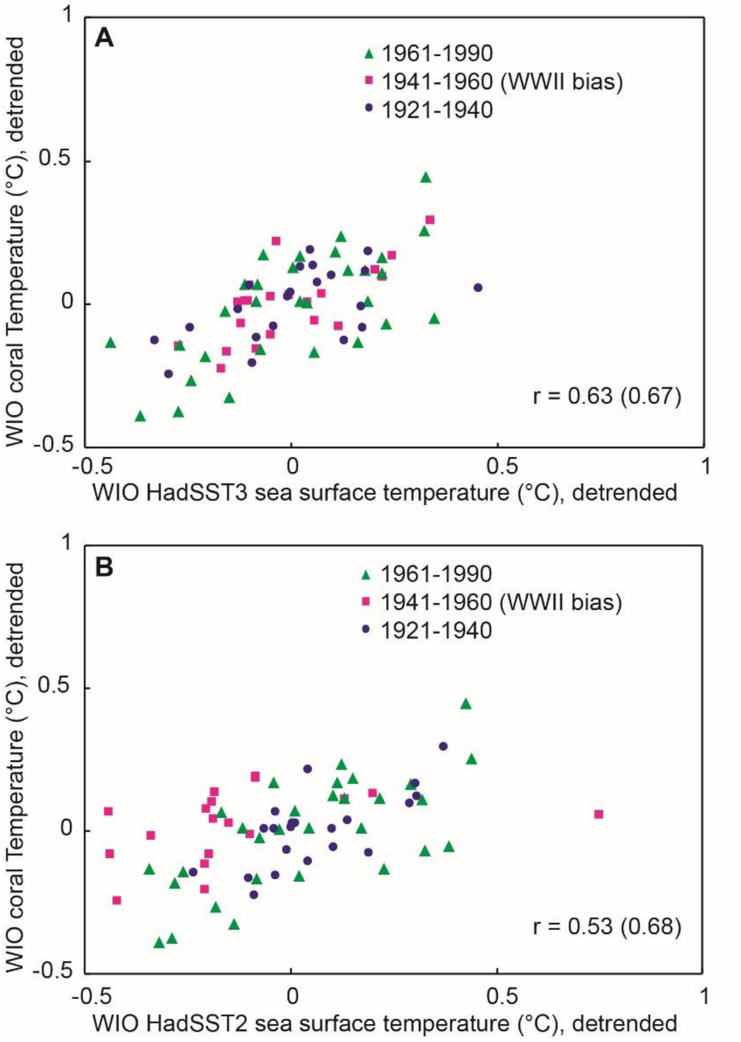

Fig. S15. WW II bias correction and coral-temperature correlation.

Scatterplots of annual mean WIO coral temperature vs. **a)** WIO HadSST3[11,12]

and **b)** WIO HadSST2[18] shown in Fig. 5 of the main manuscript after removing the linear trends. Green triangles: 1961-1990, magenta squares: 1941-1960 (WW II bias), blue dots: 1921-1940. Note the improved correlation between WIO HadSST3 and WIO coral temperature during the time period affected by the WW II bias. Correlation coefficients are calculated over the 1921-1990 period and omitting the WW II bias period from 1941-1960 (in brackets). All correlations are significant at the 1% level assuming 67 (48) degrees of freedom, N=69 (N=50).

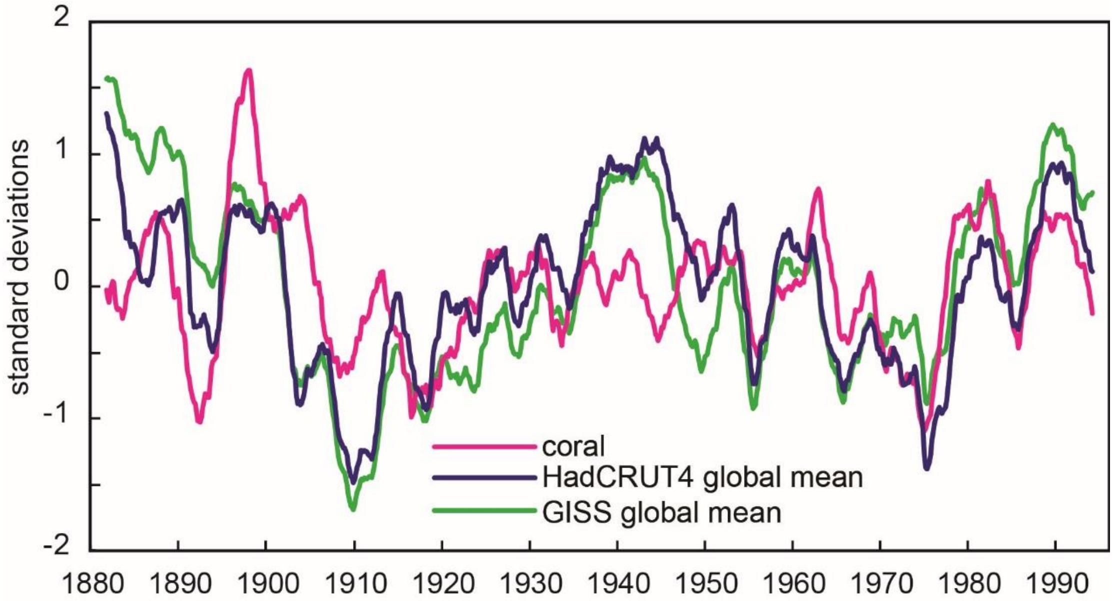

Fig. S16. Western Indian Ocean corals and global mean temperature. WIO coral temperature reconstruction (magenta) compared with global mean temperature (blue line: HadCRUT4[21]; green line: GISTEMP 1200km[13]) after removing the linear trends. Shown are 21 point moving averages of bimonthly anomalies, normalized to their standard deviation over the 1900-1995 period. The correlation between the coral and HadCRUT4 (GISTEMP) is r=0.51 (0.46), significant at the 1% level assuming 31 degrees of freedom (21 point average of bimonthly time series: n=668/21, N-2=31). Note the good agreement

between the WIO coral reconstruction and global mean temperature in the 1950-1995 period, when 5 coral cores are available and instrumental data coverage in the Western Indian Ocean is good.
